# Genome-wide investigation of the AP2/ERF gene family in tartary buckwheat (*Fagopyum Tataricum*)

**DOI:** 10.1186/s12870-019-1681-6

**Published:** 2019-02-20

**Authors:** Moyang Liu, Wenjun Sun, Zhaotang Ma, Tianrun Zheng, Li Huang, Qi Wu, Gang Zhao, Zizhong Tang, Tongliang Bu, Chenglei Li, Hui Chen

**Affiliations:** 1 College of Life Science, Sichuan Agricultural University, Ya’an, China; 2 College of Biological Industry, Chengdu University, Chengdu, Sichuan China

**Keywords:** Tartary buckwheat, *FtAP2/ERF*, Genome-wide, Fruit development, Expression patterns

## Abstract

**Background:**

*AP2/ERF* transcription factors perform indispensable functions in various biological processes, such as plant growth, development, biotic and abiotic stresses responses. The *AP2/ERF* transcription factor family has been identified in many plants, and several *AP2/ERF* transcription factors from *Arabidopsis thaliana* (*A. thaliana*) have been functionally characterized. However, little research has been conducted on the *AP2/ERF* genes of tartary buckwheat (*Fagopyum tataricum*), which is an important edible and medicinal crop. The recently published whole genome sequence of tartary buckwheat allowed us to study the tissue and expression profiles of *AP2/ERF* genes in tartary buckwheat on a genome-wide basis.

**Results:**

In this study, 134 *AP2/ERF* genes of tartary buckwheat (*FtAP2/ERF*) were identified and renamed according to the chromosomal distribution of the *FtAP2/ERF* genes. According to the number conserved domains and gene structure, the *AP2/ERF* genes were divided into three subfamilies by phylogenetic tree analysis, namely, *AP2* (15 members), *ERF* (116 members) and *RAV* (3 members). A total of 10 motifs were detected in tartary buckwheat *AP2/ERF* genes, and some of the unique motifs were found to be important for the function of *AP2/ERF* genes.

**Conclusion:**

A comprehensive analysis of *AP2/ERF* gene expression patterns in different tissues and fruit development stages by quantitative real-time PCR (qRT-PCR) showed that they played an important role in the growth and development of tartary buckwheat, and genes that might regulate flower and fruit development were preliminarily identified. This systematic analysis establishes a foundation for further studies of the functional characteristics of *FtAP2/ERF* genes and improvement of tartary buckwheat crops.

**Electronic supplementary material:**

The online version of this article (10.1186/s12870-019-1681-6) contains supplementary material, which is available to authorized users.

## Background

*AP2/ERF* transcription factors (TFs) compose one of the largest families of plant transcriptional regulation genes and regulate the signaling networks of various biological processes in plants [1, 2]. The *AP2/ERF* genes contain one or two AP2 DNA-binding domains with 60 to 70 conserved amino acid residues [3, 4]. According to the number of AP2 domains and other DNA binding domains, the *AP2/ERF* family can be divided into three subfamilies: *AP2*, *RAV*, and *ERF* gene families [5]. Among the three subfamilies, only the *AP2* family contains two conserved AP2 domains, whereas all the others contain only one AP2 domain [6]. The members of the *RAV* family include not only an AP2 domain but also a DNA-binding domain B3 that exists in other plant-specific transcription factors [7, 8]. Furthermore, according to differences in the promoter sequence of *ERF* family binding interaction genes, a categorization into *ERF* and *DREB* subfamilies can be established [9]. The *ERF* subfamily and *DREB* subfamily were further divided into six groups consisting of clades A1-A6 and clades B1-B6 in *A. thaliana* [9].

Since *AP2* transcription factors can regulate *A. thaliana* flower development [10], a large number of *AP2* genes have been identified in various plants. The TFs from the *AP2* family are mainly involved in the regulation of plant growth and development, such as flower development [11–13], leaf shape [14] and seed growth [15]. Ethylene response factors (ERFs) are located downstream of the ethylene signaling pathway, and many proteins in the *ERF* family have been identified and found to be involved in many functions, such as metabolic regulation [16–18], responses to biotic and abiotic stresses [19–21], hormone signaling [22], and plant development [23]. However, RAV TFs can regulate leaf senescence [24] and participate in biotic and abiotic stress responses [25].

Tartary buckwheat (*Fagopyrum tataricum*) is recognized as an important functional food material because it contains abundant flavonoids with antioxidant activity [26]. The essential amino acid composition of the seed protein is not only balanced but also the total content is higher than that of primary grain crops. The auxin response factor gene family has been identified from tartary buckwheat, and the potential role of *Fagopyrum tataricum ARF2* (*FtARF2*) in tartary Buckwheat fruit size has been deeply studied [27, 28]. Meanwhile, the MADS gene family has also been identified from tartary buckwheat [29]. With the increasing demand for tartary buckwheat, understanding the potential regulatory mechanism of growth and development has been a key problem. The *AP2/ERF* family plays an important role in regulating plant growth and development. At present, whole-genome identification and analysis of the *AP2/ERF* gene family have been performed in many plants, including *A. thaliana* [5], rice [30], cauliflower [31], Chinese cabbage [32], pear [33], sesame [34], and pepper [35].

However, there is no information about the *AP2/ERF* family in tartary buckwheat (*Fagopyrum tataricum*). Because of the importance of *AP2/ERF* genes in various physiological processes, it is important to systematically study the *AP2/ERF* family of tartary buckwheat. The evolutionary characteristics and tissue-specific expression of the *AP2/ERF* gene family in tartary buckwheat can be characterized by recently completed genome sequencing. In this study, we comprehensively analyzed the gene structure, motif composition, chromosomal locations, gene duplications, and expression patterns of 134 tartary buckwheat *AP2/ERF* genes and compared the evolutionary relationships with *A. thaliana*, *Beta vulgaris*, *Glycine max*, *Solanum lycopersicum*, *Vitis vinifera*, *Oryza sativa*, and *Helianthus annuus*. In addition, we studied the expression pattern of *AP2/ERF* genes in developmental stages of tartary buckwheat fruit. By global expression analysis, the participation of members of specific *AP2/ERF* family genes in different biological processes of tartary buckwheat was determined. The results of this study could provide valuable information for screening important *AP2/ERF* genes in tartary buckwheat growth and development.

## Methods

### Genes identification and classification

The largest number of *AP2/ERF* genes were found in tartary buckwheat genome downloaded from the Tartary Buckwheat Genome Project (TBGP; http://www.mbkbase.org/Pinku1/) by two BLASTP methods. The candidate genes were searched by BLASTP using a score value of ≥100 and e-value ≤ e^− 10^. Then the hidden Markov model (HMM) file from the Pfam protein family database (http://pfam.xfam.org/) corresponding to the *AP2* domain (PF00847) and the *B3* domain (PF02362) was downloaded. *AP2/ERF* genes were retrieved from the tartary buckwheat genomic database by HMMER3.0. The default parameter was determined and the cutoff set to 0.01. Using the PFAM and SMART programs to determine the existence of *AP2* core sequence and genes contains *AP2* domain were further verified by HMMER. Finally, 134 *AP2/ERF* gene models were identified in the tartary buckwheat genome for further analysis. The basic information of the identified *AP2/ERF* proteins were obtained using the tools at the ExPasy website (http://web.expasy.org/protparam/).

### Sequence analysis

The structural differences between *Fagopyrum tataricum AP2/ERF* (*FtAP2/ERF*) genes were investaged by studying the conserved motifs of the encoded *AP2/ERF* proteins. Alignment of *FtAP2/ERF* protein sequences with ClustalW default parameters. The exon-intron structure of the *FtAP2/ERF* genes were determined by Gene Structure Display Server (GSDS: http://gsds.cbi.pku.edu.cn/) and the conserved motifs of AP2/ERF proteins were evaluated by MEME online program (http:/meme.nbcr.net/meme/intro.html) [36].

### Chromosomal distribution and gene duplication of *FtAP2/ERF* genes

The method of mapping *FtAP2/ERF* genes on the chromosome of tartary buckwheat was the same as that of *Ft*ARFs genes [36]. Analysis of gene replication events using Multiple collinear scanning toolkits (MCScanX). The syntenic analysis maps of the Dual Systeny Plotter software (https://github.com/CJ-Chen/TBtools) was constructed to determine the syntenic relationship between *FtAP2/ERF* gene and *AP2/ERF* genes in other selected plants.

### Phylogenetic analysis and classification of the *FtAP2/ERF* gene family

According to the number of *AP2* conserved domain and the existence of B3 conserved domain, *FtAP2/ERF* genes were divided into different groups. The AP2/ERF protein sequences of *A. thaliana*, *Beta vulgaris*, *Glycine max*, *Solanum lycopersicum*, *Vitis vinifera*, *Oryza sativa* and *Helianthus annuus* were downloaded from the UniProt database (https://www.uniprot.org/). The phylogenetic trees were constructed by the neighbor-joining (NJ) method, the parameters refer to Liu et al. [36].

### Plant materials

Professor Wang Anhu of Xichang University gave the tartary buckwheat accessions (XIQIAO) used in this study. The XIQIAO with high rutin content were obtained by physical and chemical mutagenesis [37]. Since 2013, XIQIAO has been introduced into the College of Life Science, Sichuan Agricultural University (Lat. 29°97’ N, 102°97′ E, Alt. 580 m), Sichuan Province, China, and grown in experimental field. The materials including tartary buckwheat flowers, the fruit from three (13, 19, and 25 days after pollination, DAP) different developmental fruit stages, and the stem, root, and leaf of mature tartary buckwheat were collected in 2017. The collected samples were quickly placed in liquid nitrogen and stored at− 80 °C for further extract RNA.

### Expression analysis of *FtAP2/ERF* genes by real-time PCR

Using the tartary buckwheat (Pinku1) genome sequence database (http://www.mbkbase.org/Pinku1/) to download the corresponding sequences of *FtAP2/ERF* genes. Meanwhile, the qRT-PCR primers were designed using Primer3 software (http://frodo.wi.mit.edu/) (Additional file 1: Table S4). Analysis of spatial and temporal expression of some *FtAP2/ERF* gene by qRT-PCR. Histone H3 gene are expressed in almost all tissues with little difference in expression levels and are often used as internal reference genes. The FtH3 gene was used as an internal control, and each qRT-PCR experiment with SYBR Premix Ex Taq II (TaKaRa) was performed at least three times using a CFX96 Real Time System (Bio-Rad). The experimental data were processed by the 2^-△△CT^ method [38].

### Statistical analysis

All the data were analyzed by analysis of variance using the Origin Pro 2018b (OriginLab Corporation., Northampton, Massachusetts, USA) statistics program, and the means were compared by the least significant difference test (LSD) at significance levels of 0.05 and 0.01.

## Results

### Identification of *FtAP2/ERF* genes in tartary buckwheat

All possible AP2/ERF genes were excavated from the tartary buckwheat genome using the two BLAST methods. Because buckwheat genomes are sequenced using a genome-wide shotgun strategy, although they are located on different scaffolds, some of these *AP2/ERF* genes may be redundant. After removing the redundant and alternate forms of the same gene, 134 potential AP2/ERF proteins were identified and renamed based on their chromosomal location (Additional file 2: Table S1).

Gene characteristics included the coding sequence length (CDS), protein molecular weight (MW), isoelectric point (PI) and subcellular localization. Of the *134 FtAP2/ERF* proteins, *FtPinG0003194700.01* was the smallest protein with *71* amino acids (*213* aa) and *FtPinG0003183000.01* was the largest one with 641 amino acids (*1923* aa) (Additional file 1). The MW of the proteins ranged from 8.09 to 71.06 KDa, and the pI ranged from 4.62 (*FtPinG0005080800.01*) to 12.01 (*FtPinG0007485600.01)*. The predicted subcellular localization results showed that *111 FtAP2/ERF* proteins were located in the nuclear region, 13 *FtAP2/ERF* proteins were located in the chloroplast region, 5 *FtAP2/ERF* proteins were located in the mitochondrial region, 4 *FtAP2/ERF* proteins were located in the cytoplasmic region, and *1 Ft AP2/ERF* protein was located in the plasma membrane (Additional file 2: Table S1).

### Multiple sequence alignment, phylogenetic analysis, and classification of *FtAP2/ERF* genes

The phylogenetic relationship of FtAP2/ERF proteins was performed by multiple sequence alignment of the *AP2* domain involving approximately 60–70 amino acids and the B3 domain consisting of 100–120 residues. The sequence alignment of all *AP2/ERF* genes showed that the YRG (7th amino acid to 9th amino acid), LG (59th amino acid and 60th amino acid), AA (68th amino acid and 69th amino acid) and YD (72th amino acid and 73th amino acid) elements were highly conserved (Additional file 3: Figure S1). The WLG element (58th amino acid to 60th amino acid) was more highly conserved in the *ERF* family and *RAV* family than in the AP2 family. In the *AP2* family, WLG elements (58th amino acid to 60th amino acid) were converted into YLG elements (58th amino acid to 60th amino acid). These conserved amino acid profiles may contribute to the classification of *AP2/ERF* genes in other species. By comparing the protein structure, we can effectively predict the function of proteins [5].

**Fig. 1:**
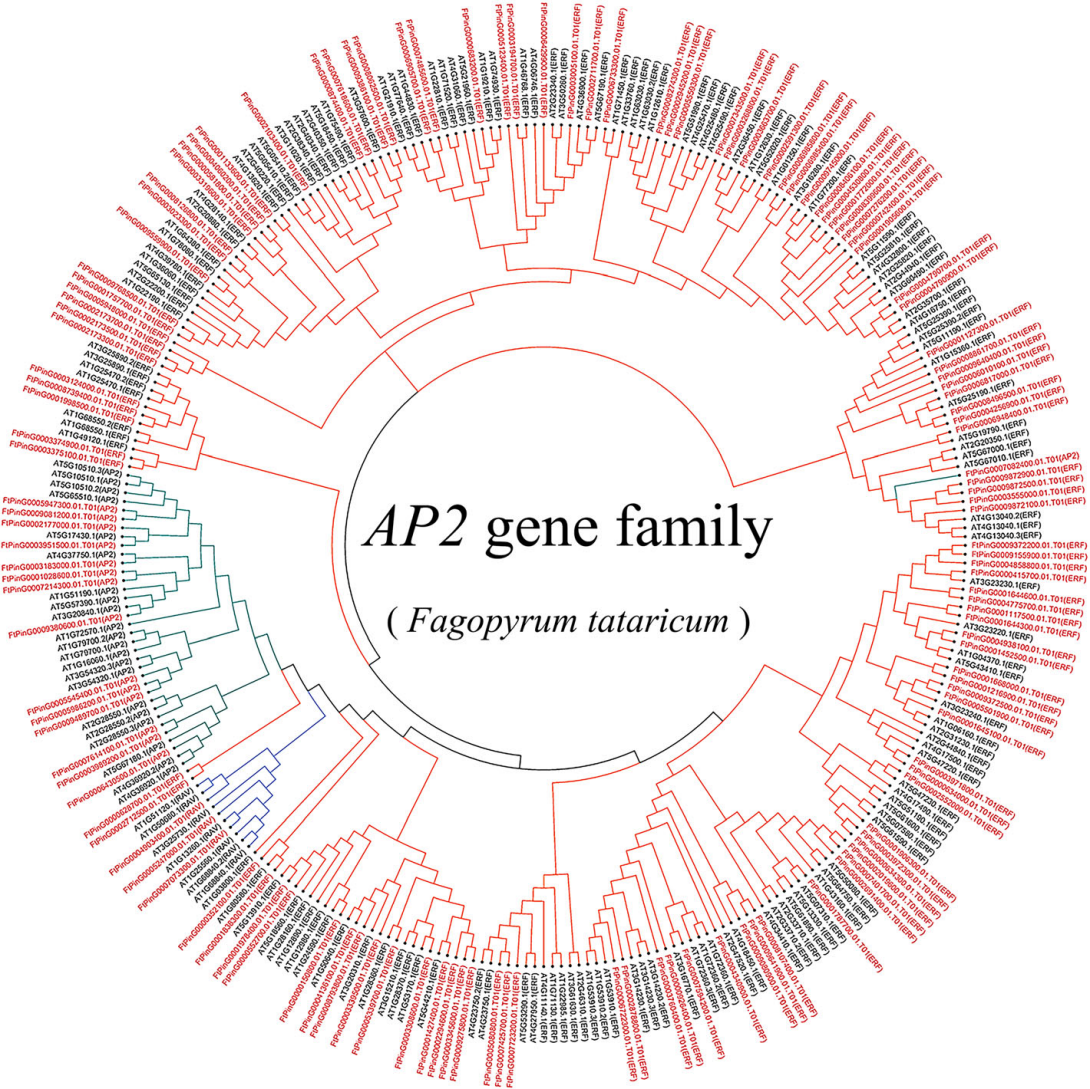
Unrooted phylogenetic tree representing the relationships among 134 *AP2/ERF* genes of tartary buckwheat and Arabidopsis. The genes in tartary buckwheat are marked in red, while those in Arabidopsis are marked in black

To explore the phylogenetic relationship of AP2/ERF proteins in tartary buckwheat, we constructed a phylogenetic tree using the neighbor-joining (NJ) method based on multiple sequence alignments of 166 *A. thaliana* AP2/ERF proteins and 134 tartary buckwheat AP2/ERF proteins. The phylogenetic distribution showed that *AP2/ERF* genes were divided into three major categories, *AP2*, *ERF*, and *RAV* (Fig. 1). Among 134 candidate *FtAP2/ERF* genes, 15 *FtAP2/ERF* genes containing two AP2/ERF domains were assigned to the AP2 family; 116 *FtAP2/ERF* genes encoding proteins containing a single AP2/ERF domain belonged to the ERF family; only 3 *FtAP2/ERF* genes encoded a single AP2/ERF domain; and a B3 domain was assigned to the RAV family (Fig. 1). Interestingly, *FtPinG0007082400* was also found to encode two AP2/ERF domains, but they were distinct from the *AP2* family and instead clustered in the *ERF* family.

### Gene structure and motif composition of the *FtAP2/ERF* gene family

**Fig. 2:**
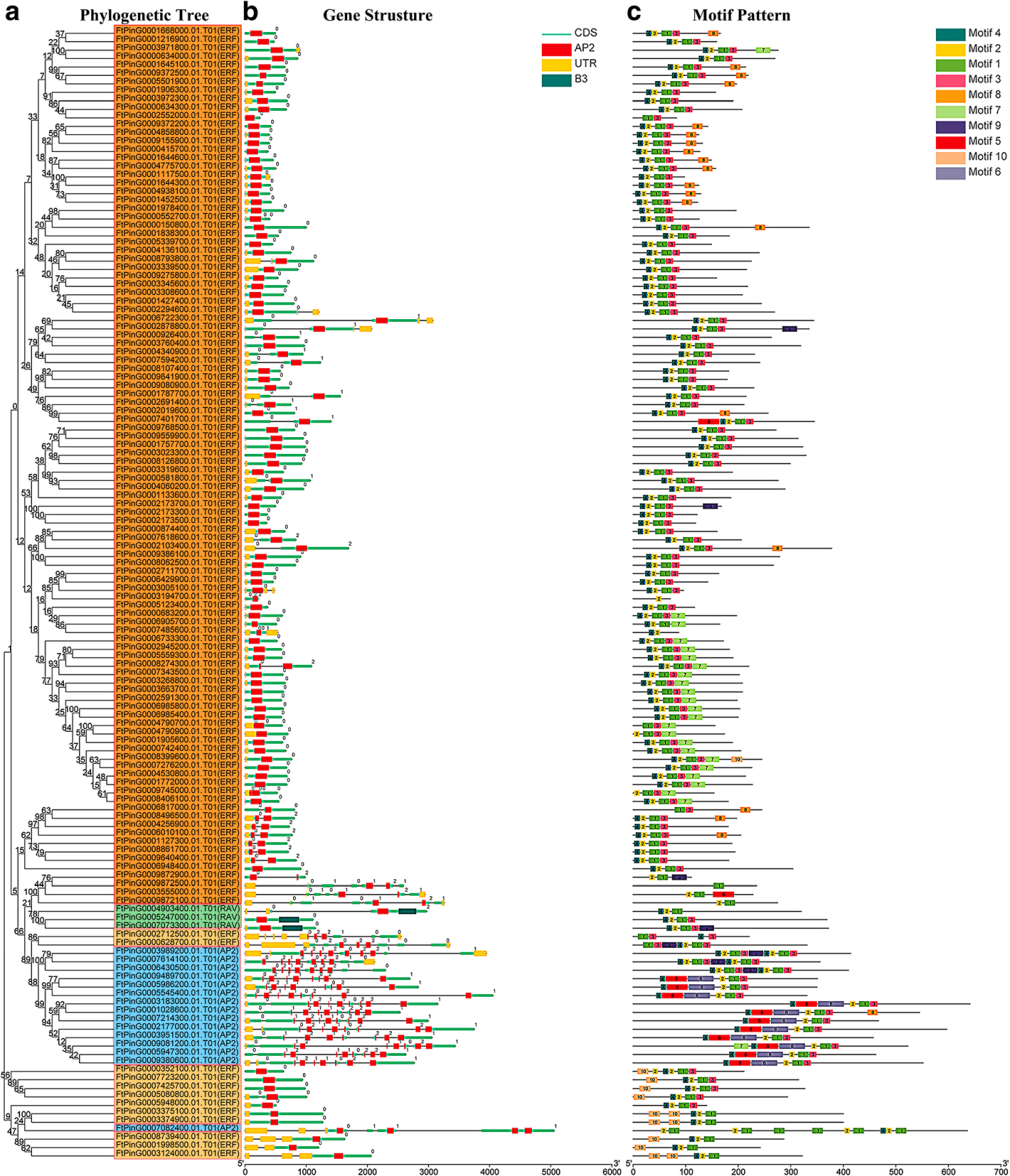
Phylogenetic relationships, gene structure and architecture of conserved protein motifs in *AP2/ERF* genes from tartary buckwheat. **a** The phylogenetic tree was constructed based on the full-length sequences of tartary buckwheat AP2/ERF proteins using Geneious R11 software. **b** Exon-intron structure of tartary buckwheat *AP2/ERF* genes. Yellow boxes indicate untranslated 5′- and 3′-regions; blue boxes indicate exons; black lines indicate introns. The AP2 domains are highlighted by red boxes and B3 domain by blackish green boxes. The number indicates the phases of the corresponding introns. **c** The motif composition of tartary buckwheat AP2/ERF proteins. The motifs, numbers 1–10, are displayed in different colored boxes. The sequence information for each motif is provided in Table S2. The protein length can be estimated using the scale at the bottom

By comparing the genomic DNA sequences of *FtAP2/ERF* genes, we obtained the intron and exon structure of *FtAP2/ERF* genes to further understand the structural composition of *FtAP2/ERF* genes (Fig. 2b). The coding sequences of all tartary buckwheat *AP2/ERF* genes were disrupted by introns, with exon numbers ranging from 1 to 9 (Fig. 2b). Excluding four exons in the *FtPinG0007082400* gene, the other members of the *AP2* subfamily contained more than 7 exons. Moreover, the number of exons was conserved in the AP2 family, although the exon positions varied. Most members of the *RAV* subfamily and *ERF* subfamily contained only one exon and the AP2 domain located in the exon region (Fig. 2b). In general, the closest members from the same subfamily had similar exon / intron structures in terms of the intron number and exon length. Further analysis showed that *FtAP2/ERF* proteins contained, at most, two characteristic regions (Fig. 2b). The N-terminal region of all *FtAP2/ERF* proteins had a highly conserved AP2 region of approximately 60–70 amino acid residues corresponding to the DNA binding region, and the *RAV* subfamily also contained the B3 region composed of 100–120 amino acids. In general, many conserved motifs can be detected in transcriptional factor protein sequences, which may be involved in activating the expression of genes as potential DNA binding sites.

The motifs of 134 *FtAP2/ERF* genes were analyzed using online MEME software to further study the characteristic regions of FtAP2/ERF proteins (Additional file 4: Table S2). According to the results of the MEME motif analysis, a schematic diagram was constructed to characterize the structure of FtAP2/ERF proteins. A total of 10 conserved motifs were found in the FtAP2/ERF proteins (Fig. 2c). Motif-1, Motif-2, Motif-3, Motif-4, and Motif-7 were found in the *AP2* domain regions, in which Motif-1, Motif-2, Motif-3, and Motif-4 were detected in almost all AP2/ERF proteins. All *ERF* subfamily genes contained Motif-1, Motif-2, Motif-3, and Motif-4, Motif-8 was detected in 17 *ERF* genes, Motif-7 in 20 *ERF* genes, Motif-10 in 5 *ERF* genes, and Motif-9 in only 3 ERF genes. In the *AP2* subfamily, 11 genes contained Motif-1 to Motif-6 and 3 *AP2* genes contained Motif-1, Motif-2, Motif-3, Motif-4, and Motif-9. The similarity in motif composition in the same subfamily indicated the conserved protein structure of a specific subfamily, and the functions of these conserved motifs must be further elucidated. The conserved motif composition and gene structure of the same subfamily were similar, thus verifying the reliability of the phylogenetic tree population classification.

### Chromosomal distribution and gene duplication and synteny analysis of *FtAP2/ERF* genes

**Fig. 3:**
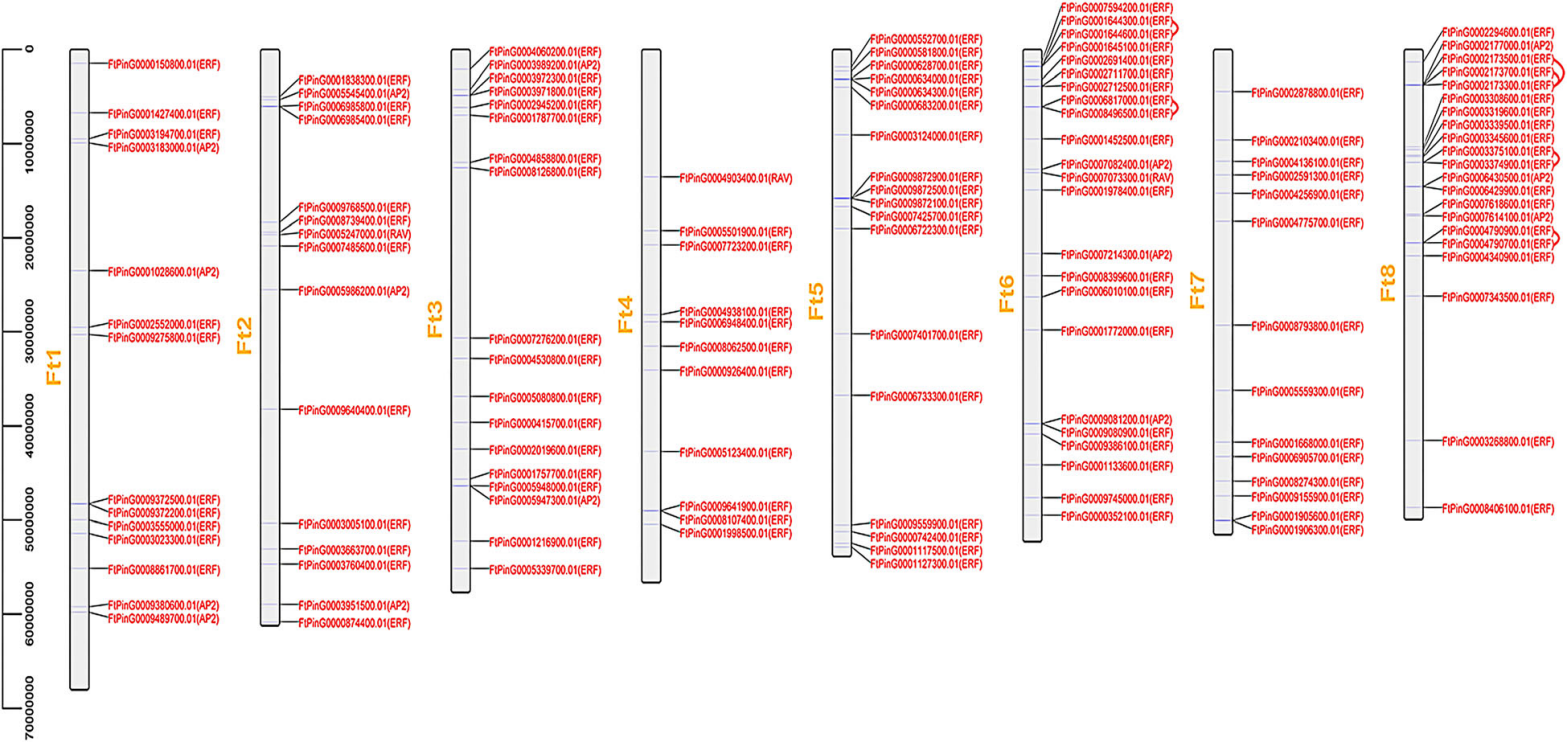
Schematic representations for the chromosomal distribution of tartary buckwheat *AP2/ERF* genes. The red lines indicate duplicated *AP2/ERF* gene pairs. The chromosome number is indicated to the left of each chromosome

Chromosome mapping of *FtAP2/ERF* genes was performed using the latest tartary buckwheat genome database. A total of 134 *AP2/ERF* TFs were unevenly distributed on eight tartary buckwheat chromosomes (Fig. 3). The largest number of *AP2/ERF* TFs was found on chromosomes 6 and 8 (23 and 21, respectively), while chromosome 4 had the smallest number of *AP2/ERF* TFs (11 genes). We found only ERF subfamily members on chromosomes 5 and 7, an absence of *AP2* subfamily members on chromosomes 4, 5 and 7, and three *RAV* subfamily members distributed on chromosomes 2, 4 and chromosome 6 (Fig. 3). Interestingly, some transcription factors with similar conserved sequences were located on the same chromosome. Similar patterns have been found in *A. thaliana* [9], *Vitis vinifera* [39] and Chinese cabbage genomes [32], which were thought to represent homologous fragments caused by ancestral polyploidy events.

**Fig. 4:**
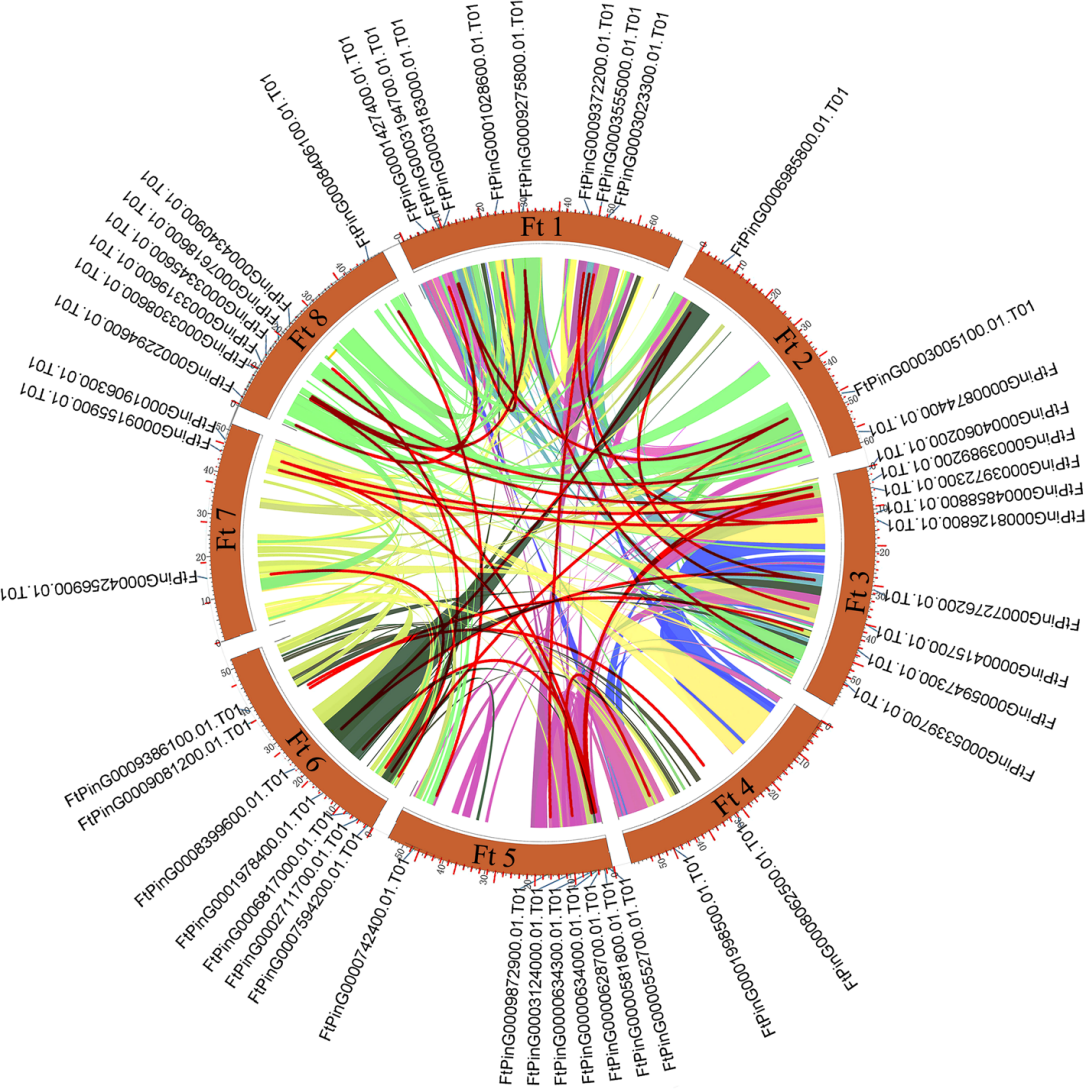
Schematic representations of the interchromosomal relationships of tartary buckwheat *AP2/ERF* genes. Colored lines indicate all syntenic blocks in the tartary buckwheat genome

In addition, we also analyzed the duplication events of *AP2/ERF* genes in the tartary buckwheat genome since gene replication plays an important role in the occurrence of novel functions and gene expansion. Chromosomal regions within the 200 kb range of two or more genes were defined as tandem replication events. Twelve *FtAP2/ERF* genes were clustered into six tandem repeat event regions in tartary buckwheat linkage group (LG) 6 and 8 (Fig. 3). LG8 had four clusters, indicating hot spots of *FtAP2/ERF* gene distribution. A pair of tandem replication genes (*FtPinG0004790900.1* and *FtPinG0004790700.1*) located on LG8 contained different motifs with other genes clustered together. In addition to tandem duplications, many pairs of segmental duplications were found in the tartary buckwheat chromosomes (Fig. 4). Analyses of homologous protein families is of great significance in establishing the kinship of species and predicting the function of new protein sequences. Many homologous genes were present on different chromosomes in tartary buckwheat, supporting the high conservation of the *AP2/ERF* gene family (Fig. 4). In brief, based on the above results, some *FtAP2/ERF* genes might be produced by gene replication, and these replication events were the main driving force of *FtAP2/ERF* evolution.

### Evolutionary analysis of *FtAP2/ERF* genes and several different species

To deduce the evolutionary relationship of *AP2/ERF* genes, phylogenetic tree analysis was performed for seven dicotyledonous plants (*A. thaliana*, *Beta vulgaris*, *Glycine max*, *Solanum lycopersicum*, *Vitis vinifera*, *Helianthus annuus* and *Tartary buckwheat*) and a monocotyledonous plants *Oryza sativa*. The *AP2/ERF* family of tartary buckwheat contained three subfamilies: *AP2*, *ERF* and *RAV*. To explore the evolutionary relationship of each gene, a phylogenetic tree analysis was performed between each subfamily of tartary buckwheat and other plant members of the same subfamily. Simultaneously, the motifs of the corresponding member proteins were determined.

**Fig. 5:**
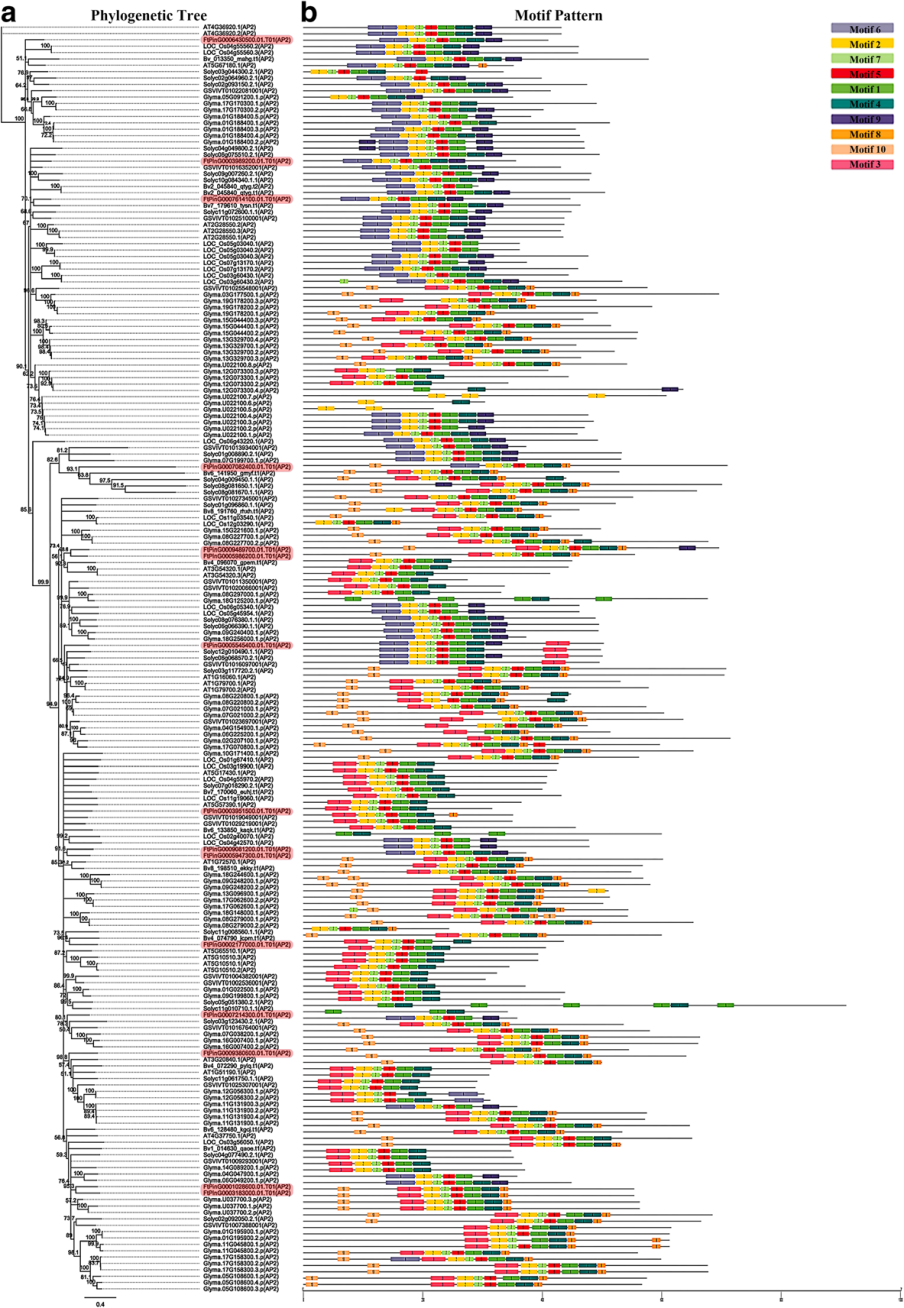
Phylogenetic relationships and motif compositions of AP2 proteins from six different plant species. Left panel: An unrooted phylogenetic tree constructed using Geneious R11 with the neighbor-joining method. Right panel: Distribution of conserved motifs in AP2 proteins. The differently colored boxes represent different motifs and their position in each AP2 protein sequence

**Fig. 6:**
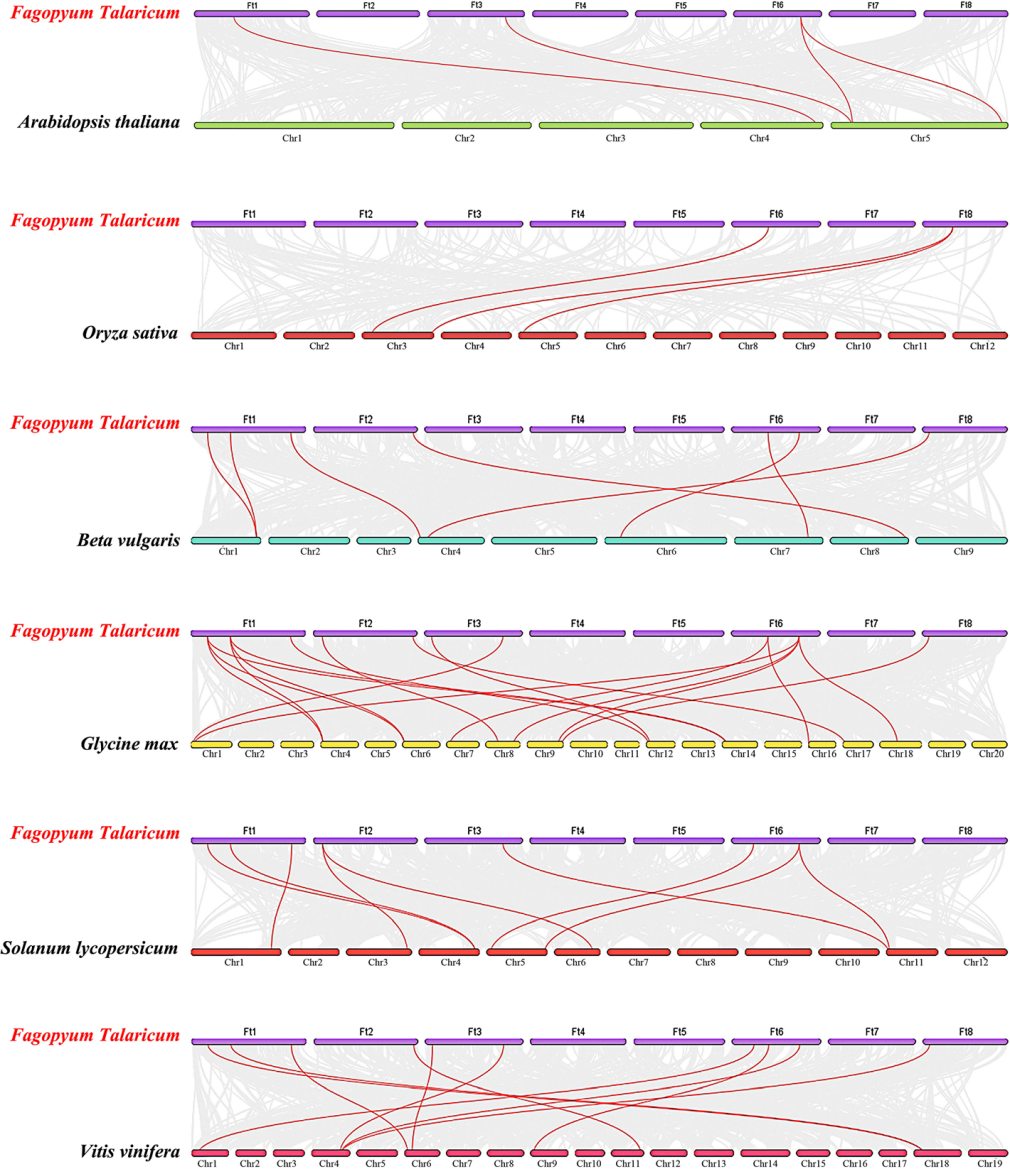
Synteny analysis of *AP2* genes between tartary buckwheat and six representative plant species. Gray lines in the background indicate the collinear blocks within tartary buckwheat and other plant genomes, while red lines highlight syntenic *AP2* gene pairs

From Fig. 5a, we can see that most members of the tartary buckwheat *AP2* subfamily were clustered with *Beta vulgaris* (5 members), followed by *A. thaliana* (4 members), *Solanum lycopersicum* (3 members), and *Glycine max* (2 members). A total of 10 conserved motifs were detected in the protein sequences of *AP2* subfamily members in all plants (Fig. 5b). Almost all members contained Motif-1, Motif-2, Motif-4, Motif-5 and Motif-7. Additionally, *AP2* members with a similar relationship in different plants had the same motif composition. Based on previous studies, we performed a syntenic analysis of *AP2* genes in six dicotyledonous plants (Tartary buckwheat*, A. thaliana*, *Beta vulgaris*, *Glycine max*, *Solanum lycopersicum* and *Vitis vinifera*) and a monocotyledonous plant *Oryza sativa* to speculate on the evolutionary origin of *AP2* genes. The *AP2* subfamily genes in tartary buckwheat have homology to reference plants, and the most syntenic conservation was observed among *Glycine max* (18 orthologous gene pairs distributed on LG1, LG4 LG6, LG7, LG8, LG9, LG12, LG14, LG6L, G17 and LG18), *Vitis vinifera* (10 orthologous gene pairs distributed on LG1, LG4, LG6, LG9, LG11 and LG18), and *Solanum lycopersicum* (9 orthologous gene pairs distributed on LG1, LG3, LG4, LG5, LG6 and LG11) (Fig. 6). In the syntenic analysis of *AP2* genes of tartary buckwheat and *Glycine max*, *FtPinG0009081200.01* was found to be associated with at least three syntenic gene pairs, suggesting that *FtPinG0009081200.01* might play an important role in *AP2* subfamily evolution (Additional file 5: Table S3).

**Fig. 7:**
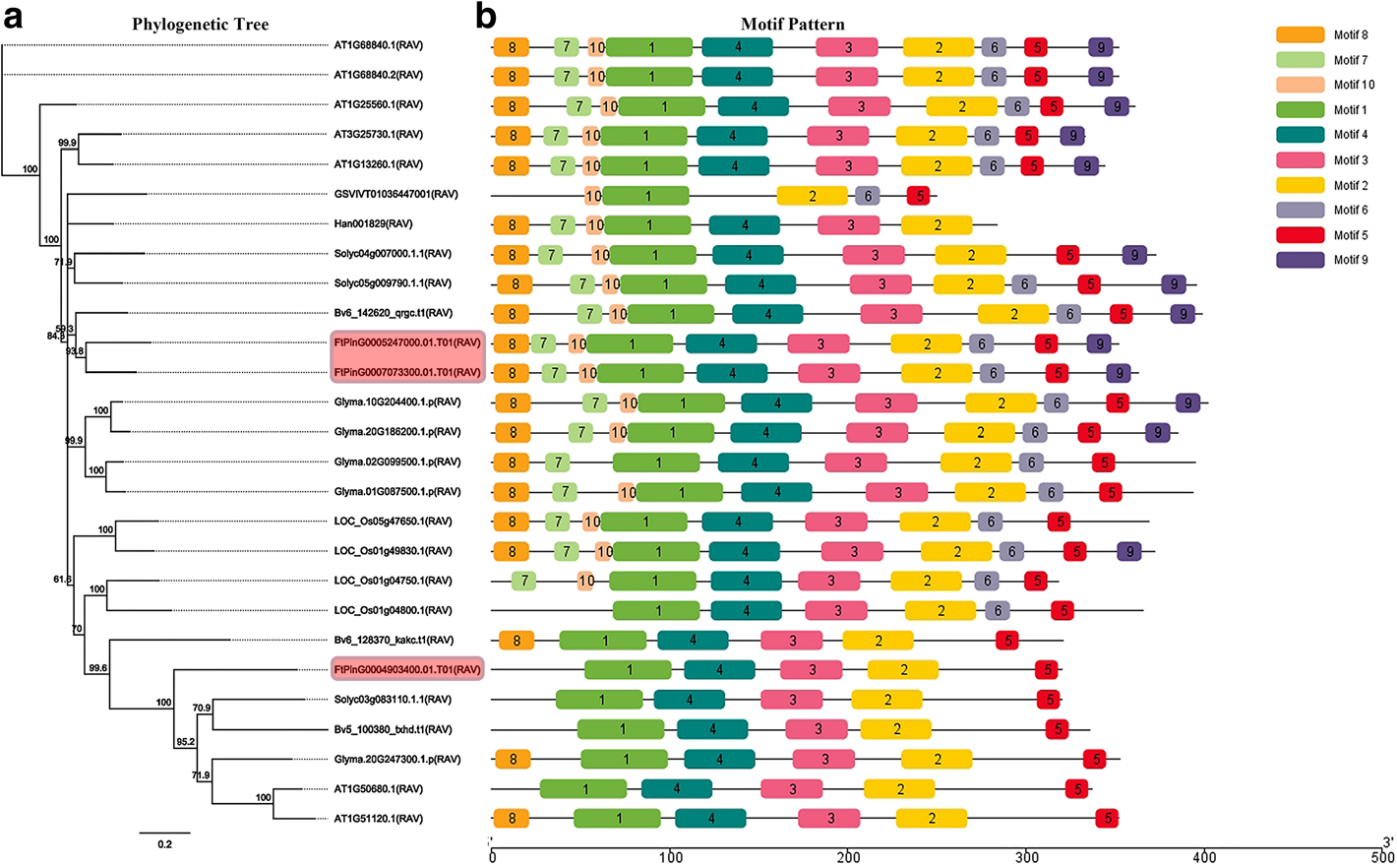
Phylogenetic relationships and motif compositions of RAV proteins from seven different plant species. Left panel: An unrooted phylogenetic tree constructed using Geneious R11 with the neighbor-joining method. Right panel: Distribution of conserved motifs in RAV proteins. The differently colored boxes represent different motifs and their positions in each RAV protein sequence

We used the same phylogenetic tree method to analyze the clustering relationship between *FtRAV* genes and the *RAV* genes of other plants. The phylogenetic tree results showed that three *FtRAV* genes were closely related to RAV genes in *Beta vulgaris* (2 members) and *Solanum lycopersicum* (1 member) (Fig. 7a). The protein sequences of the *RAV* genes also showed 10 conserved motifs, and all the members contained Motif-1, Motif-2, Motif-3, Motif-4 and Motif-5 (Fig. 7b). Moreover, the members of the same branch of the phylogenetic tree had the same motif composition.

**Fig. 8:**
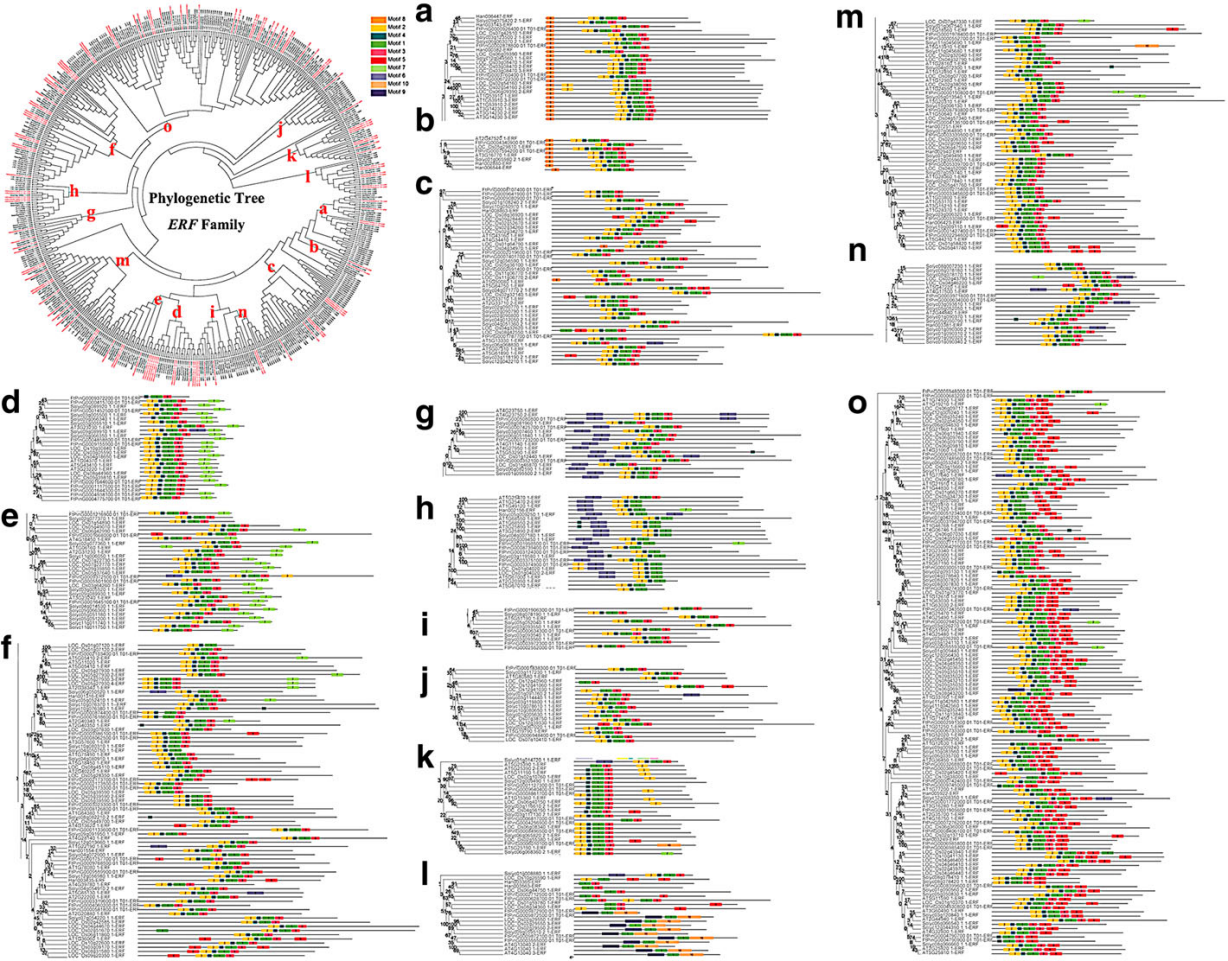
Phylogenetic relationships and motif compositions of ERF proteins from five different plant species. Left panel: An unrooted phylogenetic tree constructed using Geneious R11 with the neighbor-joining method. Right panel: Distribution of conserved motifs in ERF proteins. The differently colored boxes represent different motifs and their positions in each ERF protein sequence

**Fig. 9:**
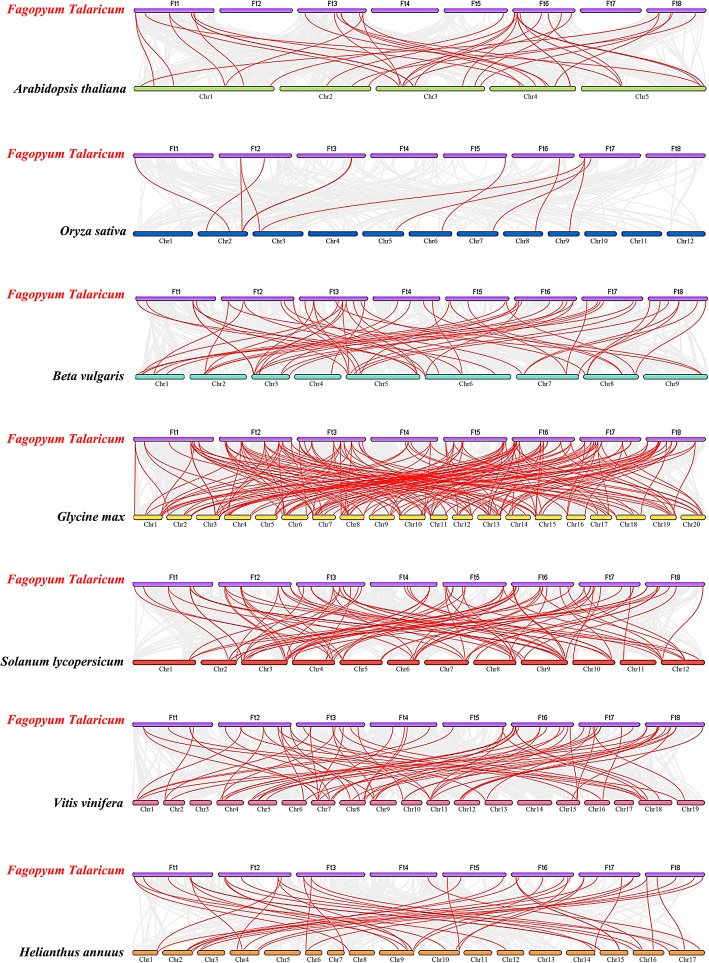
Synteny analysis of *ERF* genes between tartary buckwheat and seven representative plant species. The gray lines in the background indicate the collinear blocks within tartary buckwheat and other plant genomes, while the red lines highlight the syntenic *ERF* gene pairs

The *ERF* subfamily of tartary buckwheat contains many members. The phylogenetic tree constructed using the *FtERF* genes and *ERF* members from three dicotyledonous plants, *A. thaliana*, *Helianthus annuus* and *Solanum lycopersicum,* and a monocotyledonous plant *Oryza sativa,* indicated that the FtERFs proteins were divided into 15 groups (Fig. 8). We detected 10 motifs in the *ERF* subfamily of all plants. All the members, excluding group-h and group-l, contained Motif-1, Motif-2, Motif-3, and Motif-4, and the genes that clustered together contained similar motifs. Group-a and group-b specifically contained Motif-8, group-o specifically Motif-5, and group-g and group-h specifically Motif-6 (Fig. 8). Based on the syntenic results, the syntenic relationships between *FtERF* and *ERF* genes from other plants were very obvious, and according to the relationships, the order was *Glycine max* (198 orthologous gene pairs distributed throughout all LGs), *Solanum lycopersicum* (101 orthologous gene pairs distributed throughout all LGs), *Vitis vinifera* (73 orthologous gene pairs distributed throughout all LGs except the LG3, LG14, and LG17), *Beta vulgaris* (59 orthologous gene pairs distributed throughout all LGs), *Helianthus annuus* (44 orthologous gene pairs distributed throughout all LGs except LG5, LG11, and LG13), *A. thaliana* (37 orthologous gene pairs distributed throughout all LGs), and *Oryza sativa* (12 orthologous gene pairs distributed on LG2, LG3, LG5, LG6, LG7 LG8, and LG9), respectively (Fig. 9). Some *FtERF* genes were found to be associated with at least two pairs of homologous genes (especially between tartary buckwheat and *Glycine max ERF* genes), such as *FtPinG0003005100.01* and *FtPinG0002711700.01*, which suggested that these genes might play an important role in the *ERF* subfamily during evolution (Additional file 5: Table S3). The syntenic analysis provided reliable evidence to support and validate the previous phylogenetic groupings and motif distribution. In general, these data indicated that the tartary buckwheat *AP2/ERF* gene family was highly conserved and the tartary buckwheat *AP2/ERF* genes were closer to the *Glycine max* genes than to the *A. thaliana* genes. The *AP2/ERF* genes might have evolved from the common ancestor in different plants.

### Expression patterns of *FtAP2/ERF* genes in different plant tissues

To investigate the physiological roles of *FtAP2/ERF* genes, qRT- PCR was performed to detect the tissue-specific expression of each *AP2/ERF* gene. The accumulation of transcriptional products of 15 *AP2* genes, 3 *RAV* genes and 43 *ERF* genes in root, stem, leaf, flower, and fruit was evaluated. The results showed that the transcriptional abundance of *FtAP2/ERF* genes varied greatly in different tissues and organs, suggesting that the *FtAP2/ERF* genes had multiple functions in tartary buckwheat growth and development.

**Fig. 10:**
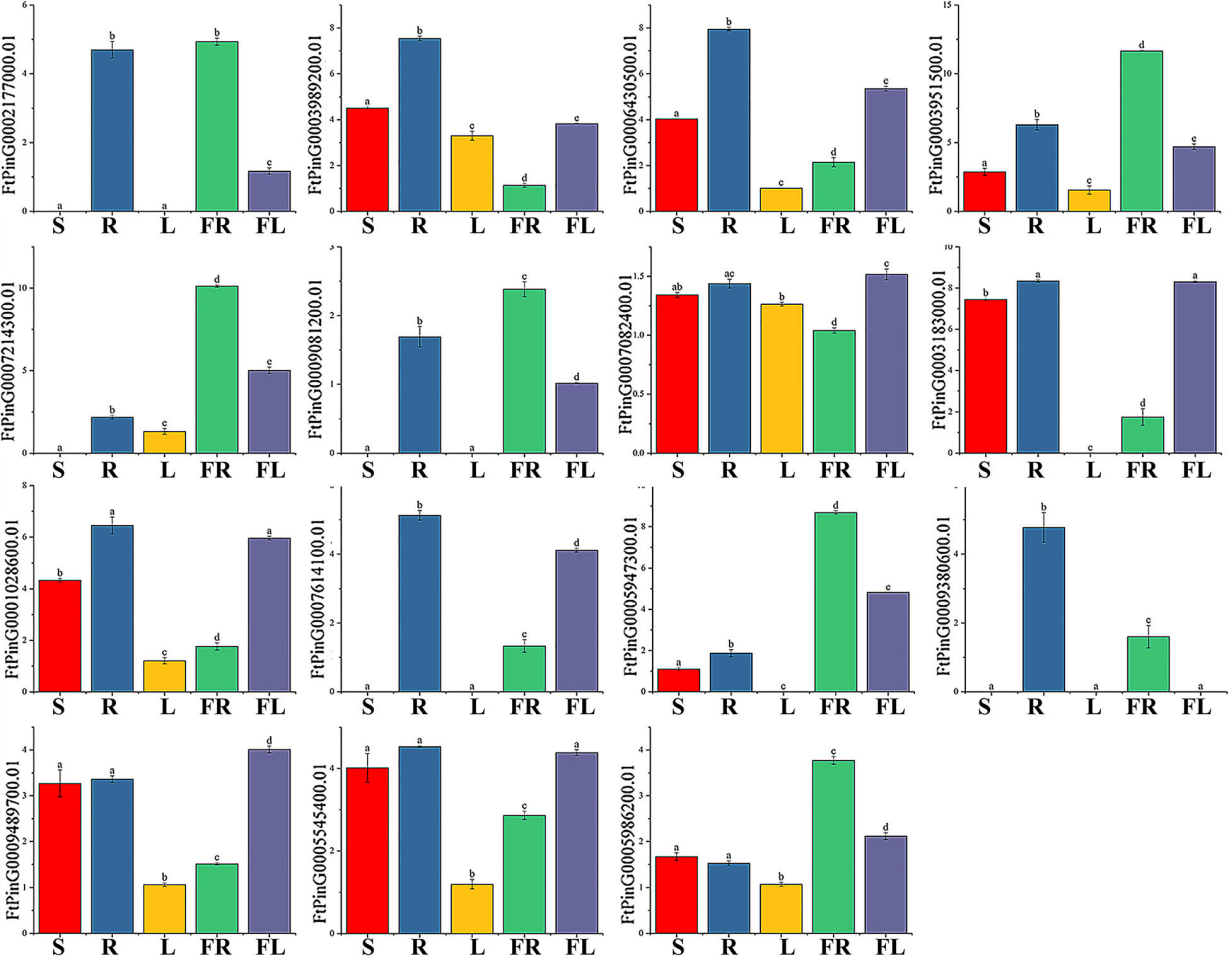
Tissue-specific gene expression of 15 tartary buckwheat *AP2* genes. The expression patterns of 15 tartary buckwheat *AP2* genes in flower, leaf, root, stem and fruit tissues were examined by qPCR. Error bars were obtained from three measurements. Small letter(s) above the bars indicate significant differences (α = 0.05, LSD) among the treatments

In the *AP2* subfamily, eight genes (*FtPinG0003989200.01/6430500.01/3951500. 01/7082400.01/ 1,028,600.01/9489700.01/5545400.01/5986200.01*) were expressed in all tissues, five genes (*FtPinG0002177000.01/7214300.01/9081200.01/5947300.01/5986200.01)* had the highest expression level in fruit, two genes (*FtPinG0007082400.01/9489700.01)* had the highest expression in flowers, and only three genes (*FtPinG0007214300.01/5947300.01/5986200.01)* were expressed at higher levels in reproductive organs than in other tissues (Fig. 10). Simultaneously, we studied the correlation of the *FtAP2* gene expression pattern in tartary buckwheat roots, stems, flowers, leaves and fruits (Additional file 6: Figure S2). Most of the *FtAP2* genes were positively correlated, and the *FtAP2* genes *(FtPinG0006430500.01* and *FtPinG0007614100.01/1028600.01*; *FtPinG0003951500.01* and *FtPinG0009081200.01*; *FtPinG0007214300.01* and *FtPinG0005986200.01/5947300.01*; *FtPinG0003183000.01 and FtPinG0005545400.01/9489700.01/7614100.01/1028600.01*) that were significantly correlated were found to be positively correlated (Additional file 6: Figure S2).

**Fig. 11:**
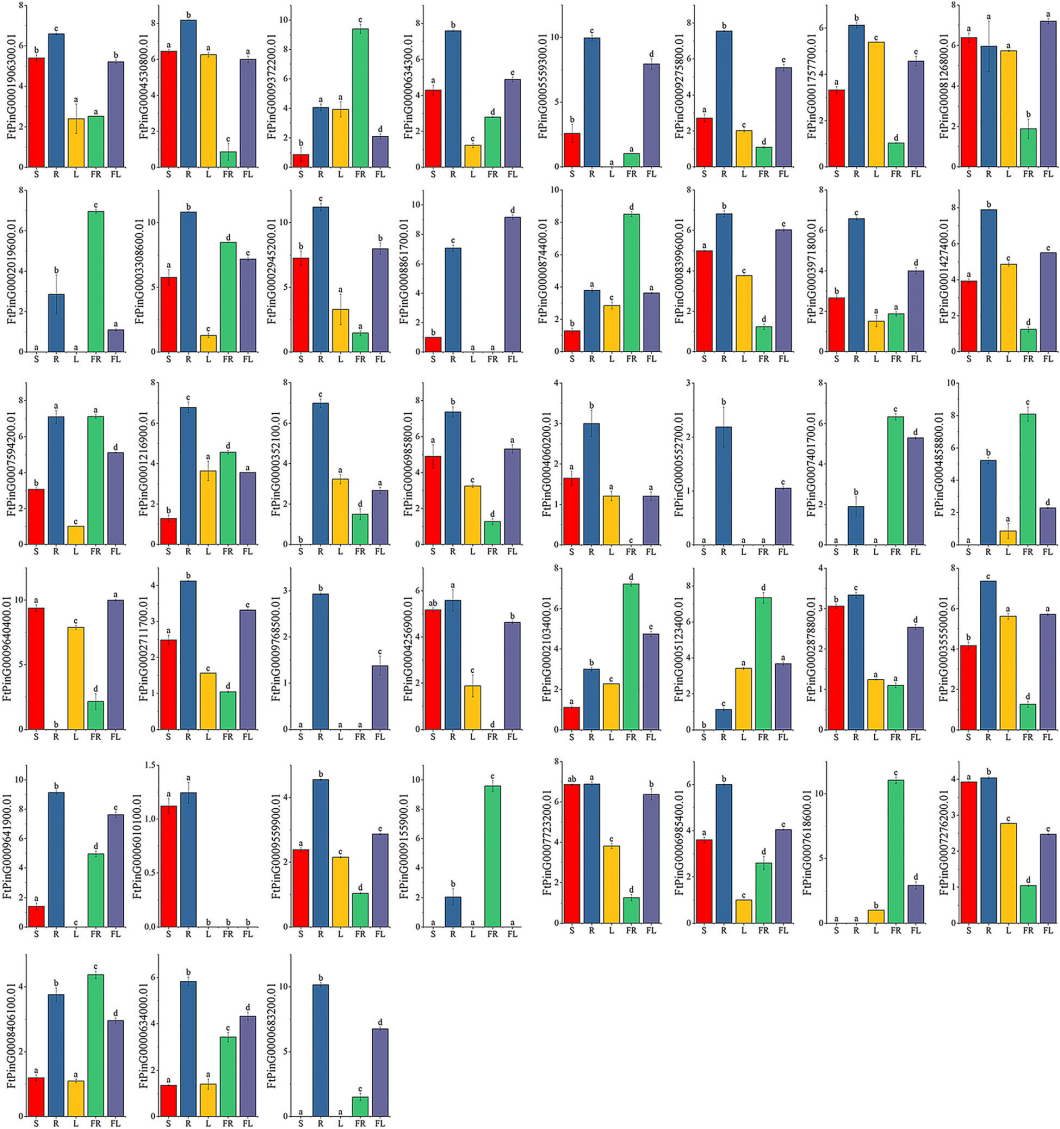
Tissue-specific gene expression of 43 tartary buckwheat *ERF* genes. The expression patterns of 43 tartary buckwheat *ERF* genes in flower, leaf, root, stem and fruit tissues were examined by qPCR. Error bars were obtained from three measurements. Small letter(s) above the bars indicate significant differences (α = 0.05, LSD) among the treatments

The *ERF* gene structures in tartary buckwheat and in *A. thaliana*, *Glycine max*, *Solanum lycopersicum* and *Helianthus annuus* were analyzed by phylogenetic tree analysis (Fig. 8). The gene structure in each group was similar, indicating the performance of potentially similar functions. The functions of many *ERF* genes in *A. thaliana* have been identified, such as *AT3G15210.1*, which negatively regulates ethylene and abscisic acid (ABA) responses. Therefore, we selected a total of 43 genes from each group with a similar evolutionary relationship to *A. thaliana ERF* (*AtERF*) genes for tissue-specific expression analysis. Among the 43 selected *ERF* genes, 60.47% were expressed in all tissues, 67.44% had the highest expression level in root, eleven (*FtPinG0009372200.01/2019600.01/874400.01/401700.01/4858800.01/2103400.01/5123400.01/9155900.01/7618600.01/8406100.01/7594200.01)* had the highest expression in fruit, and three (*FtPinG0008861700.01/ 0008126800.01/ 9640400.01)* had the highest expression in flower (Fig. 11). The correlation analysis of *ERF* subfamily gene expression in different tissues revealed a strong correlation between the genes, and most of the genes were positively correlated (Additional file 7: Figure S3). The expression level of *FtPinG0009372200.01* was highest in fruit, which was significantly positively correlated with other genes (*FtPinG0009155900.01*, *FtPinG0004858800.01*, *FtPinG0000874400.01*, and *FtPinG0002019600.01)* with a high expression level in fruit. Similarly, *FtPinG0001906300.01* was significantly positively correlated with other genes (*FtPinG0006985400.01, FtPinG0007723200.01,* and *FtPinG0002878800.01,* among others) that were highly expressed in root (Additional file 7: Figure S3).

**Fig. 12:**
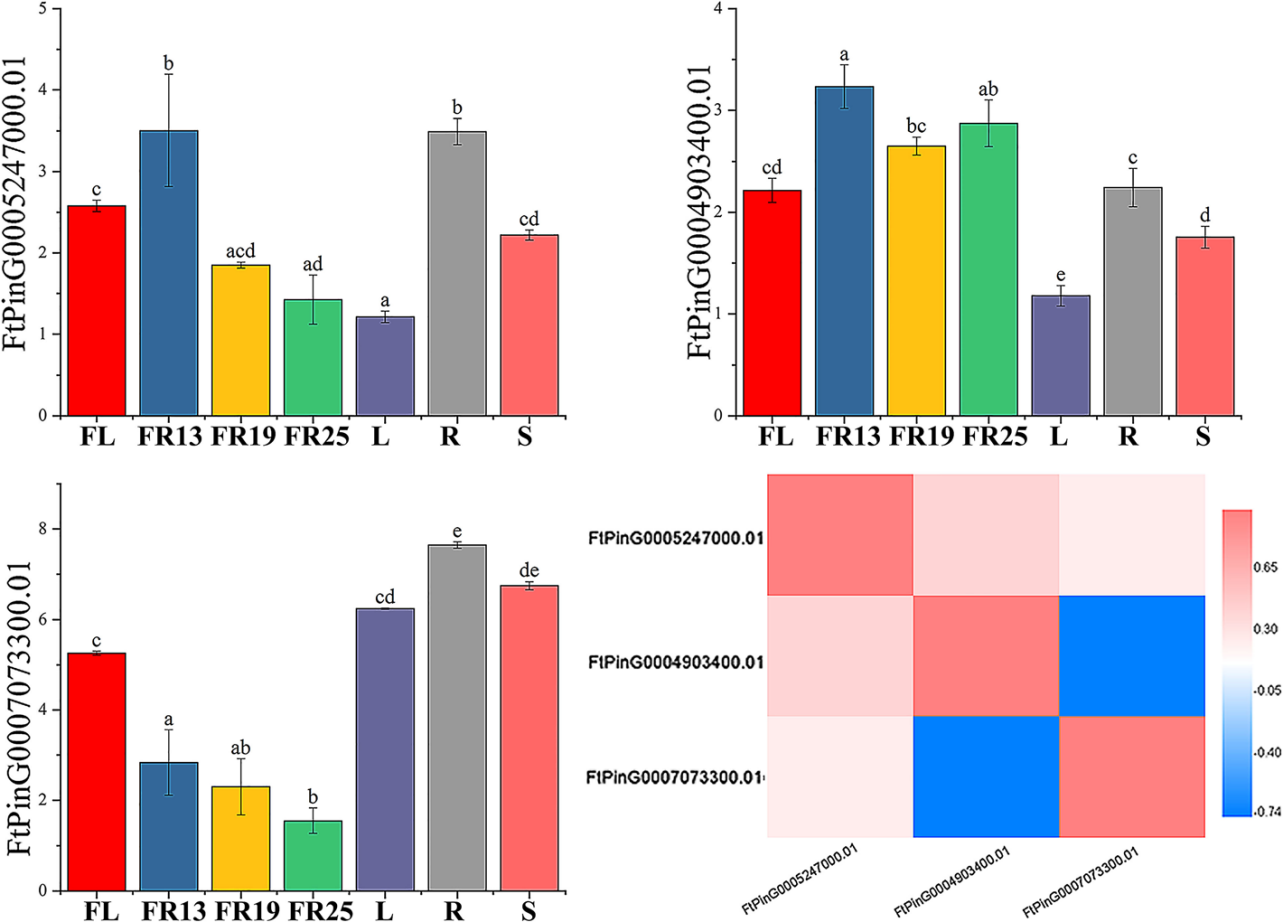
Tissue-specific gene expression and correlation between the gene expression of 3 tartary buckwheat *RAV* genes. The expression patterns of 3 tartary buckwheat *RAV* genes in flower, leaf, root, stem and fruit tissues were examined by qPCR. Error bars were obtained from three measurements. Small letter(s) above the bars indicate significant differences (α = 0.05, LSD) among the treatments. Positive number: positively correlated; negative number: negatively correlated

There were only three members of the *RAV* subfamily, among which *FtPinG0005247000.01* had the highest expression level in roots and the lowest expression level in leaves, *FtPinG0004903400.01* had the highest expression level in fruits and the lowest expression level in leaves, and *FtPinG0007073300.01* had the highest expression level in roots and the lowest expression level in fruits (Fig. 12).

### Differential expression of *FtAP2/ERF* genes during fruit development of tartary buckwheat

Ethylene is a simple but very important plant hormone. It is involved in seed germination, plant flowering, fruit maturation, organ senescence, and shedding, among other processes [40]. *ERF* family genes located downstream of the ethylene signaling pathway are important transcription factors that regulate the ethylene biosynthesis pathway. Therefore, we can systematically study the expression of *FtAP2/ERF* genes at different stages of fruit development (green fruit stage, discoloration stage and initial maturity stage) and find some genes that potentially regulate fruit maturation and development.

**Fig. 13:**
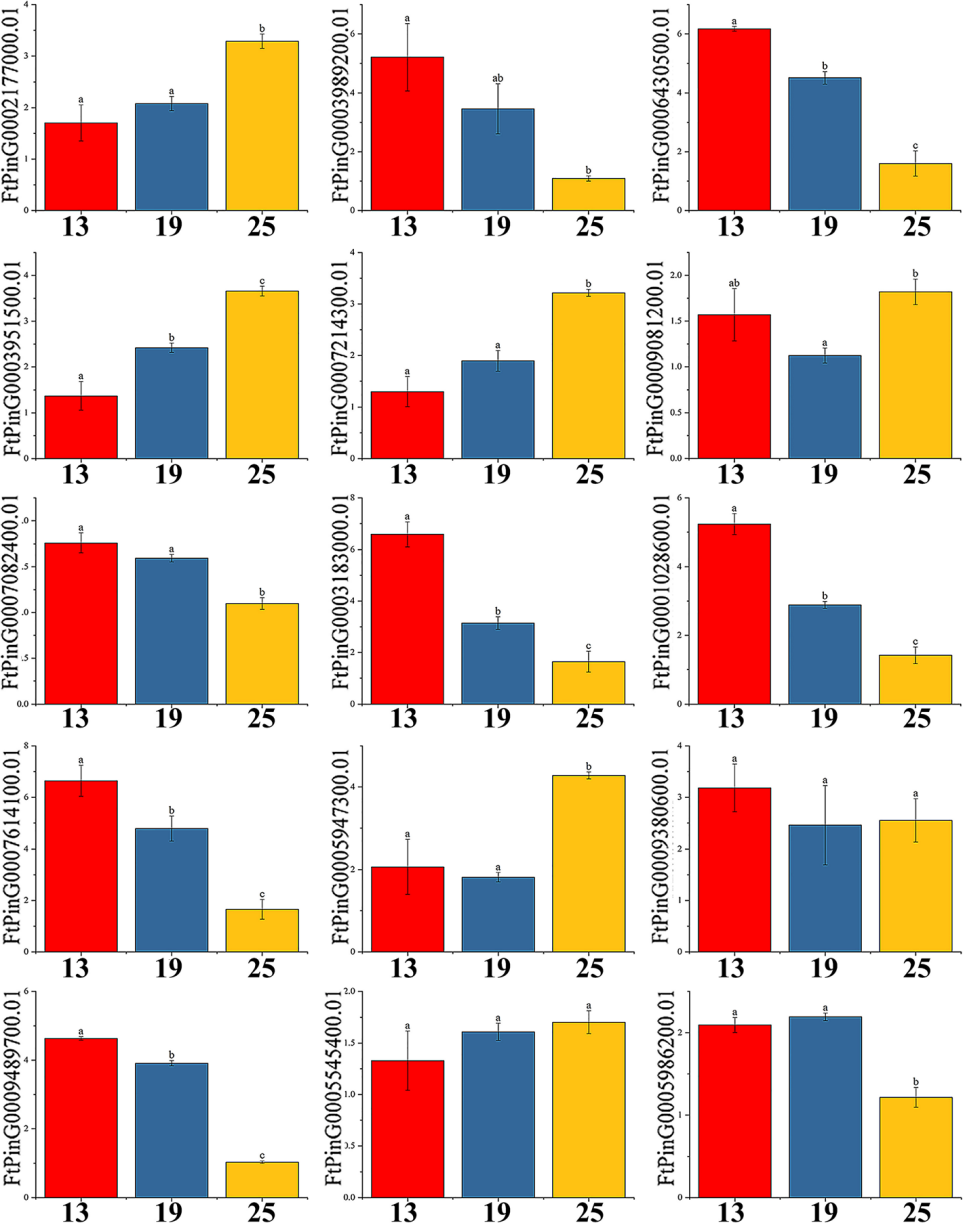
Gene expression of 15 tartary buckwheat *AP2* genes during fruit development. The expression patterns of 15 tartary buckwheat *AP2* genes in the fruit development stage were examined using a qPCR assay. Error bars were obtained from three measurements. Small letter(s) above the bars indicate significant differences (α = 0.05, LSD) among the treatments

The expression patterns of *FtAP2/ERF* tartary buckwheat fruits at different developmental stages (green fruit stage, discoloration stage and initial maturity stage) were different. In the *AP2* subfamily, all the genes were expressed in three stages. With the development of the fruit, the expression levels of eight genes (*FtPinG0003989200.01/6430500.01/7082400.01/3183000.01/28600.01/761400.01/9380600.01/9489700.01)* gradually decreased and those of six genes (*FtPinG0002177000.01 /3951500.01 /7214300.01 /9081200.01 /5947300.01 /5545400.01)* gradually increased, but only one gene (*FtPinG0005986200.01)* was expressed at the highest level in the discoloration stage (Fig. 13). Concurrently, we studied the correlation of *AP2* gene expression patterns with tartary buckwheat fruit development and the correlation of each gene during this process. As shown in Additional file 8: Figure S4, only *FtPinG0003951500.01* was significantly positively correlated with fruit development, while the *FtAP2* genes *(FtPinG0002177000.01* and *FtPinG0007214300.01*; *FtPinG0003989200.01* and *FtPinG0007614100.01/6430500.01; FtPinG0006430500.01* and *FtPinG0007614100.01; FtPinG0007082400.01* and *FtPinG0009489700.01)* were found to be significantly positively correlated.

**Fig. 14:**
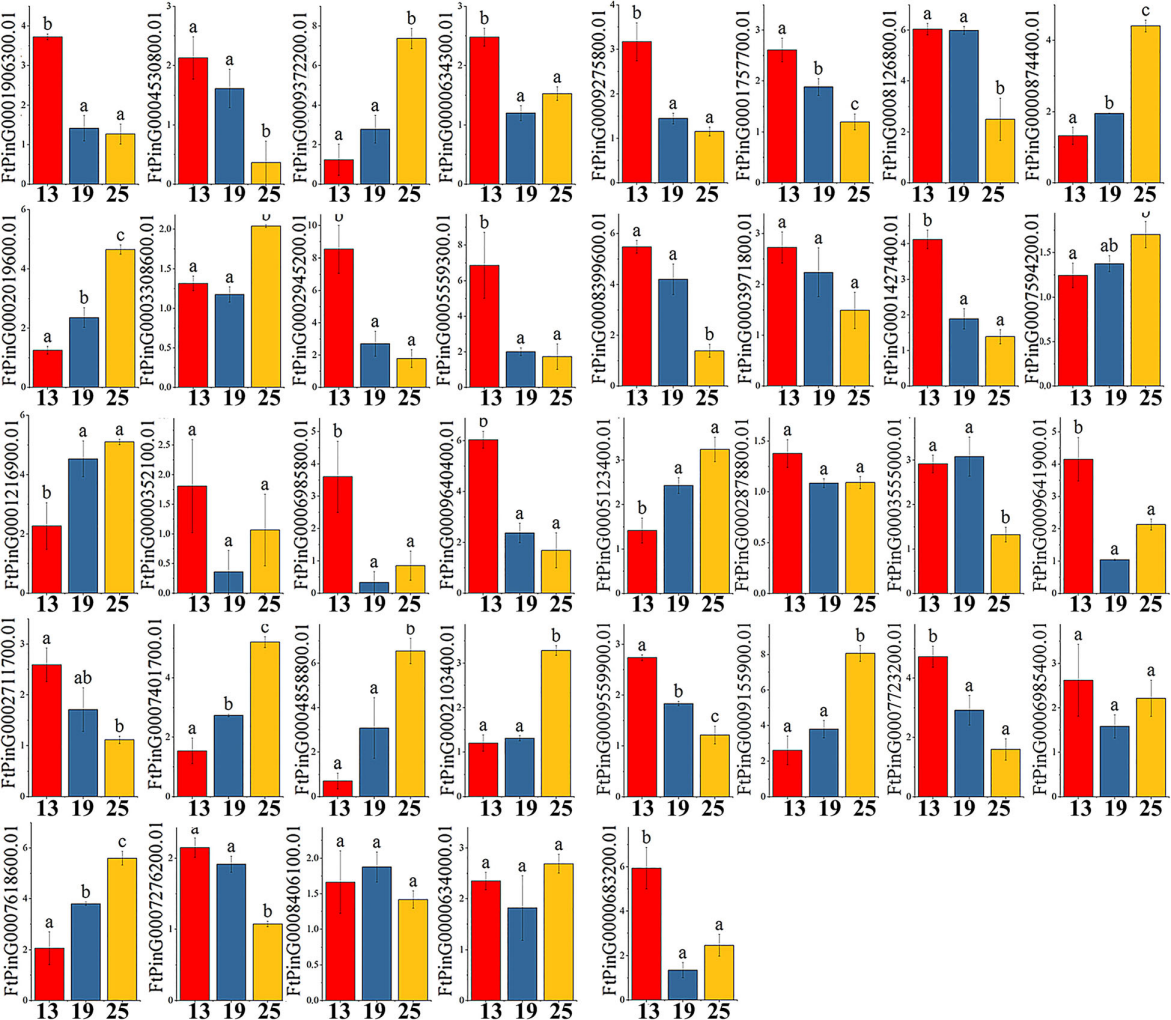
Gene expression of 43 tartary buckwheat *ERF* genes during fruit development. The expression patterns of 43 tartary buckwheat *ERF* genes in the fruit development stage were examined by qPCR. Error bars were obtained from three measurements. Small letter(s) above the bars indicate significant differences (α = 0.05, LSD) among the treatments

By analyzing the expression of *ERF* subfamily genes during three fruit developmental stages (green fruit stage, discoloration stage and initial maturity stage), we found that all the selected *ERF* genes were expressed in the fruit development stage. Among the eleven genes with the highest expression level in fruit, we found that only *FtPinG0008406100.01* was expressed at the highest level during the discoloration stage; the expression of all the other genes gradually increased throughout the fruit development stage (Fig. 14). During fruit development, the expression level of *FtPinG0008126800.01* and *FtPinG0009640400.01* gradually decreased (expressed at the highest level in flowers), while those of most genes that were highly expressed in roots also decreased with fruit development (Fig. 14). Based on the correlation analysis of *ERF* subfamily gene expression levels during different fruit development periods and of each gene during this process, we found that *FtPinG0007618600.01 and FtPinG0005123400.01* were significantly positively correlated with fruit development, while there was a significant positive correlation among *FtPinG0009372200.01* and other genes (*FtPinG0009155900.01*, *FtPinG0007594200.01 and FtPinG0000874400.01*) with high expression levels in fruit (Additional file 9: Figure S5).

In the *RAV* subfamily, the expression levels of two genes (*FtPinG0005247000.01* and *FtPinG0007073300.01)* in the three stages of fruit development (green fruit stage, discoloration stage and initial maturity stage) gradually decreased, and the expression level of only *FtPinG0004903400.01* was lowest in the discoloration stage (Fig. 12).

## Discussion

The *AP2/ERF* gene family plays an important role in various physiological processes, and it has been extensively studied in many plants, such as *A. thaliana* [9], *Vitis vinifera* [41], castor bean [42], and peach [43]. However, few studies have investigated the *AP2/ERF* genes in tartary buckwheat. In the current study, a search for *AP2/ERF* genes in the tartary buckwheat genome resulted in the identification of 134 members, including 116 *ERF* family members, 15 *AP2* family members and 3 *RAV* family members. Previous studies have revealed similar numbers of *AP2/ERF* genes in other plants, such as *Vitis vinifera* with 132 *AP2/ERF* genes, including 109 *ERF* members, and peach with 130 *AP2/ERF* members, including 104 *ERF* genes. Concurrently, the genome size of the three plants was different, with 475 Mb in *Vitis vinifera*, 265 Mb in peach and 489 Mb in tartary buckwheat, thus showing that the number of *AP2/ERF* superfamily members was relatively stable and that there was no absolute correlation with genome size.

Structural analysis of the *ERF* subfamily showed that 79.3% of *FtERF* genes had no introns, while the number of introns in the *AP2* subfamily genes ranged from 3 to 9 (Fig. 2b). The gene structure of *AP2/ERF* genes in tartary buckwheat was similar to that of *AP2/ERF* genes in *Hordeum vulgare L* [44]. The difference between the structure of *AP2* subfamily and *ERF* subfamily genes supported a vast differentiation in the genome evolution. The domains and motifs of transcription factors are often related to protein interaction, transcriptional activity and DNA binding [45]. Motif analysis showed that most of the *AP2/ERF* genes of tartary buckwheat contained Motif-1, Motif-2, Motif-3, and Motif-4, which were related to the AP2 domain (Fig. 2c). Motif-7, Motif-8 and Motif-10 were specifically detected in different groups of the *ERF* subfamily, indicating that they play an important role in this subfamily. These results suggest that although some motifs of the *AP2/ERF* family genes were highly conserved, the new evolutionary motifs might perform new functions in some plants, and the functions of these new evolutionary motifs require further verification.

Duplications of individual genes, chromosomal fragments, or entire genomes have long been considered a major source of evolution, including new gene functions and expression patterns [46]. Our study showed that most duplicated *FtERF* genes were expressed in different tissues / organs, suggesting that these genes had specific or redundant cellular functions. Evidence for differences between duplicate genes can be inferred from the expression patterns of the *FtPinG0004790900.01* and *FtPinG0004790700.01* genes. *FtPinG0004790900.01* was highly expressed in stem, but *FtPinG0004790700.01* was not expressed (Additional file 10: Figure S6). The composition and location of their motifs are identical (Fig. 8), and therefore we speculate that the reason for their expression level differences might be due to the mutation of genes involved in the process of replication, which leads to a loss of function of *FtPinG0004790700.01*. In addition, functional differences may lead to differences in gene pair expression patterns. For example, the mRNA abundance of *FtPinG0001644300.01* peaked in flower, but *FtPinG000*1644600.01 was highly expressed in root (Additional file 10: Figure S6). *FtPinG000*1644600.01 contains motif-7, but *FtPinG0001644300.01* does not (Fig. 8); thus, we speculate that the alteration of the motifs of the two genes during replication may be one of the causes of the functional differences.

The functions of genes can be preliminarily predicted by analysis of the gene expression patterns [47]. Tissue-specific expression profiles indicated that most of the *AP2* subfamily genes (53.33%) (Fig. 10), *ERF* subfamily genes (60.47%) (Fig. 11) and *RAV* subfamily genes (100%) (Fig. 12) were expressed in all tissues. However, 46.7 and 67.44% of the genes in the *AP2* subfamily and *ERF* subfamily were expressed at higher levels in roots, and similar results have been found in other plants [32, 42]. Based on the phylogenetic tree, we found that *AP2* genes (*FtPinG0002177000.01*, *FtPinG0009081200.01*, and *FtPinG0005947300.01*) that were highly expressed in tartary buckwheat fruit were clustered together (Fig. 1). *FtPinG000*2177000.01 was highly expressed not only in buckwheat fruit but also in roots (Fig. 10). Exploration of the evolutionary relationship between these *AP2* genes and other *AP2* genes in other plants revealed a similar evolutionary relationship of *FtPinG0002177000.01* and *AT5G10510.1* (*AIL6*) and identical motif compositions (Fig. 5). Moreover, *AIL6* was identified as a gene that promotes shoot growth and floral meristem initiation in *A. thaliana*, and thus this provides a direction for us to further verify the functionality of *FtPinG0002177000.01* [48]. Plants accumulate triacylglycerol in their seeds to provide carbon and energy for seedling growth. An interesting *AP2* gene, *AtWRI1* (*At3g54320*), was identified in *A. thaliana*, which was highly expressed in seeds. It can not only regulate the biosynthesis of plant oil but also promote seed germination. The *FtPinG0005986200.01* was clustered with *AtWRI1*, and their tissue-specific expression patterns were similar. Thus, it is necessary to further validate whether *FtPinG0005986200.01* can also promote seed germination. In *A. thaliana*, the expression level of *ANT (AT1G72570.1)*, a member of the *AP2* subfamily, was high in young tissues such as inflorescence and siliques but low in leaves and stems, which could promote the development of floral organs [49]. The expression pattern of *FtPinG0005947300.01* in tartary buckwheat was similar to that of *AT1G72570.1* (Fig. 10), and, as shown in Fig. 5, the evolutionary relationship of *FtPinG0005947300.01* was closely to *AT1G72570.1* and the motif composition in their core regions was very consistent. Therefore, we need to further verify whether *FtPinG0005947300.01* possesses a function similar to *AT1G72570.1* and plays an important role in regulating the growth and development of flowers and other organs.

Fruit development is divided into five stages: organogenesis, expansion, maturation, ripening, and senescence [50]. Tartary buckwheat seeds can be divided into five stages from flowering to maturation: young fruit stage (0–8 DAP), green fruit stage (8–14 DAP), discoloration stage (14–22 DAP), initial maturity stage (22–26 DAP), and mature stage (26–30 DAP), and the accumulation of plant hormones and nutrients differs in each stage [27]. Ethylene can promote fruit ripening, and ethylene signaling plays an important role in fruit development and the plant stress response [51]. The respiration rate of climacteric fruits such as tomato increases rapidly during ripening, accompanied by a rise in ethylene content [52]. In addition, the ethylene content also increases prior to veraison of nonclimacteric fruits such as grape [53]. Studies have shown that *AP2* genes not only regulate the seed quality and yield by changing the number and size of embryonic cells, but also the growth and development of flower organs [54–56], while *ERF* genes control the final steps of ethylene signal transduction by binding to the GCC box in the promoter region of pathogen-related genes, thus affecting fruit maturation [57]. Fruit size has been shown to be related to the initial state of the embryo, while the final size of tartary buckwheat fruit is related to the division of embryonic cells. Additionally, fruit size reaches its maximum from the green fruit stage to the initial maturity stage, so the tartary buckwheat fruit size was determined during the early stage of development [27, 58]. In this study, an *AP2* gene, *FtPin0009489700.01,* was shown to possess the high expression level in all tissues, but highest in fruits (Fig. 10). Moreover, it had the highest expression level in early fruit development (green fruit stage) (Fig. 13). Therefore, we can try to further verify the role of *FtPin0009489700.01* in plant growth and development from the point of effect of *FtPin0009489700.01* on fruit size in the future. The transcription factor *AtERF4*, which can negatively regulate ethylene and ABA responses, was isolated from *A. thaliana*. Phylogenetic analysis indicated a similar relationship between *FtPinG0003308600.01* and *AT3G15210.1* (*AtERF4*), and the motif composition of the genes was identical (Fig. 8). Therefore, we speculated that *FtPinG0003308600.01* might also regulate the responses of ethylene and ABA. Interestingly, we observed increasing contents of ABA in the development of buckwheat fruits (discoloration stage to initial maturity stage) [27] and of *FtPinG0003308600.01* in development stages of fruits (Additional file 8: Figure S4); thus, *FtPinG0003308600.01* might be a positive regulator of the response of ethylene and ABA, necessitating further experimental verification. In general, the *AP2/ERF* family genes were considered to play an indispensable role in fruit formation and development.

## Conclusions

This is the first comprehensive analysis of *AP2/ERF* genes in tartary buckwheat with the goal of elucidating the evolution, expression patterns and possible functions of *AP2/ERF* genes in tartary buckwheat. These findings provide insights for predicting the functions of *AP2/ERF* genes in flower and seed development, and the comprehensive analysis of the results was helpful for screening genes for further functional identification and for genetic improvement of agronomic traits of tartary buckwheat.

## Additional files

Additional file 1: **Table S4.** The primer sequences of qRT-PCR. (XLS 38 kb)

Additional file 2: **Table S1.** List of the 134 *FtAP2/ERF* genes identified in this study. (XLS 258 kb)

Additional file 3: **Figure S1.** Alignment of multiple *FtAP2/ERF* and selected AP2 domain amino acid sequences. (DOCX 4133 kb)

Additional file 4: **Table S2.** Analysis and distribution of conserved motifs in tartary buckwheat AP2/ERF proteins. (XLS 41 kb)

Additional file 5: **Table S3.** One-to-one orthologous relationships between tartary buckwheat and other plants. (XLS 106 kb)

Additional file 6: **Figure S2.** The correlation between the gene expression of *FtAP2s*. Positive number: positively correlated; negative number: negatively correlated. Red numbers indicate a significant correlation at the 0.05 level. (DOCX 1124 kb)

Additional file 7: **Figure S3.** The correlation between the gene expression of *FtERFs*. Positive number: positively correlated; negative number: negatively correlated. Red numbers indicate significant correlation at 0.05 levels. (DOCX 1348 kb)

Additional file 8: **Figure S4.** The correlation between the gene expression of *FtAP2s* during fruit development. Positive number: positively correlated; negative number: negatively correlated. Red numbers indicate significant correlation at 0.05 levels. (DOCX 911 kb)

Additional file 9: **Figure S5.** The correlation between the gene expression of *FtERFs* during fruit development. Positive number: positively correlated; negative number: negatively correlated. Red numbers indicate significant correlation at 0.05 levels. (DOCX 1390 kb)

Additional file 10: **Figure S6.** Tissue-specific gene expression of 3 tandem duplication tartary buckwheat ERF genes. The expression patterns of 3 tandem duplication tartary buckwheat ERF genes in flower, leaf, root, stem and different stage fruit tissues were examined by qPCR. Error bars were obtained from three measurements. Small letter(s) above the bars indicate significant differences (α = 0.05, LSD) among the treatments. (DOCX 238 kb)

## Abbreviations

*A. thaliana*: *Arabidopsis thaliana*
ABA: Abscisic acid
AtERF: *A. thaliana* ERF
CDS: Coding sequence
DAP: Days after pollination
ERFs: Ethylene response factors
Ft: *Fagopyrum tataricum*
LG: Linkage group
MV: Molecular weight
PI: Isoelectric point
qRT-PCR: Quantitative real-time PCR
TFs: Transcription factors
