# Supplementary figures and images for: Genome-wide investigation of the AP2/ERF gene family in tartary buckwheat (Fagopyum Tataricum)
Source: BMC Plant Biol. 2019 Feb 20;19:84. doi: 10.1186/s12870-019-1681-6 (PMC6381666; doi:10.1186/s12870-019-1681-6)

**Figure S1**


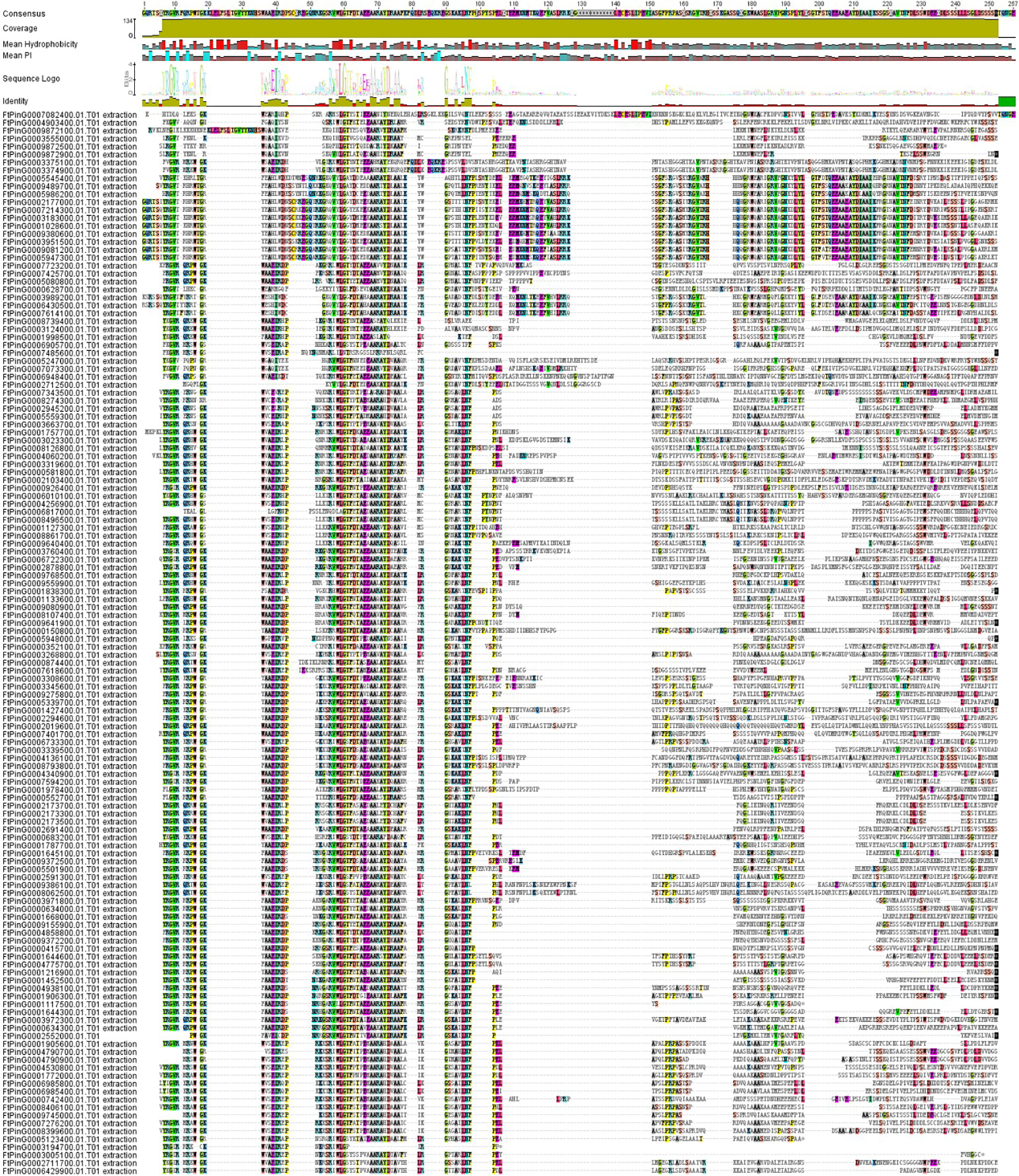

Supplement: Supplementary file 3 — Figure S1. Alignment of multiple FtAP2/ERF and selected AP2 domain amino acid sequences. (DOCX 4133 kb) [file 12870_2019_1681_MOESM3_ESM.docx]

**Figure S2**

**
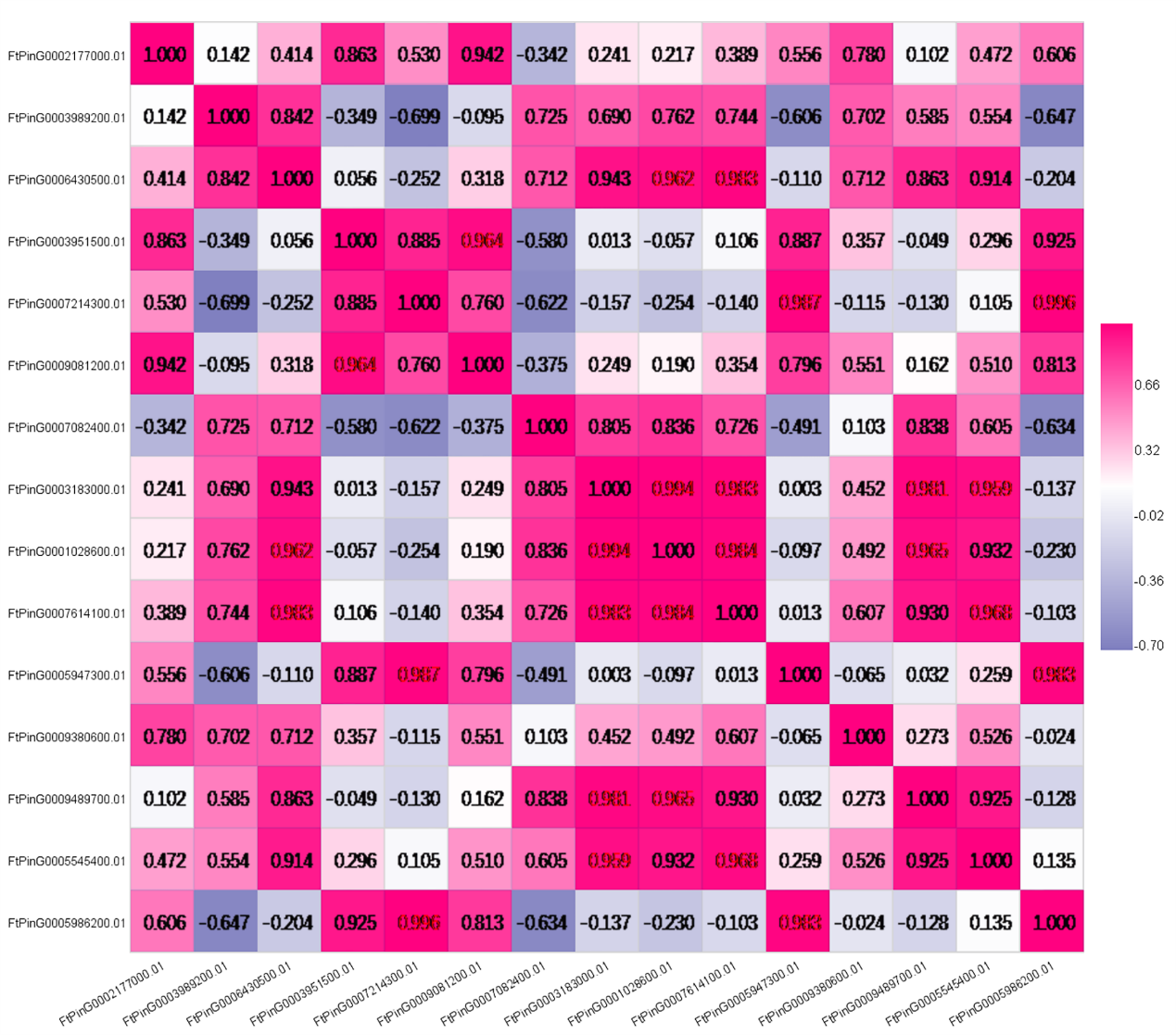
**

Supplement: Supplementary file 6 — Figure S2. The correlation between the gene expression of FtAP2s. Positive number: positively correlated; negative number: negatively correlated. Red numbers indicate a significant correlation at the 0.05 level. (DOCX 1124 kb) [file 12870_2019_1681_MOESM6_ESM.docx]

**Figure S3**

**
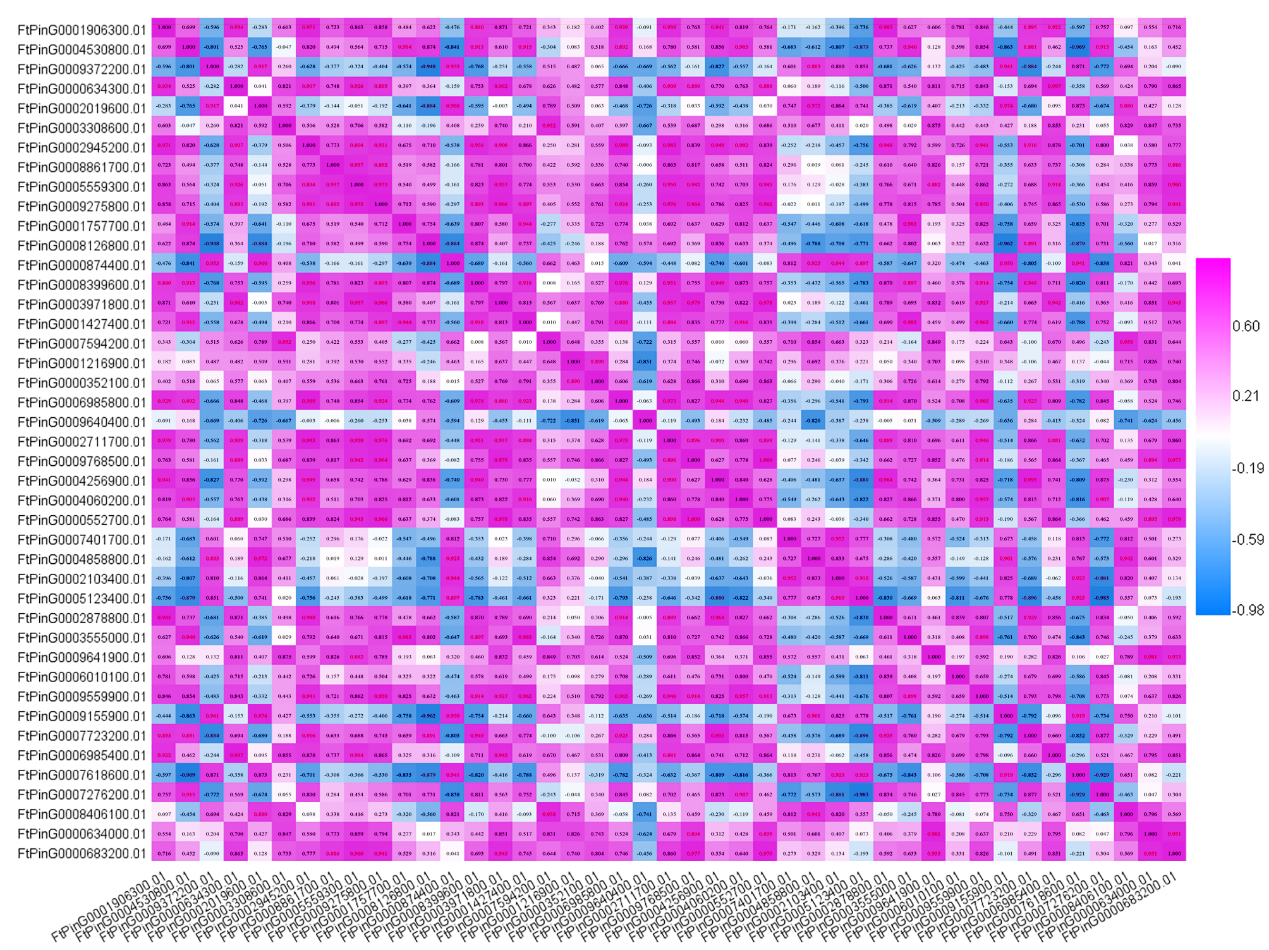
**

Supplement: Supplementary file 7 — Figure S3. The correlation between the gene expression of FtERFs. Positive number: positively correlated; negative number: negatively correlated. Red numbers indicate significant correlation at 0.05 levels. (DOCX 1348 kb) [file 12870_2019_1681_MOESM7_ESM.docx]

**Figure S4**

**
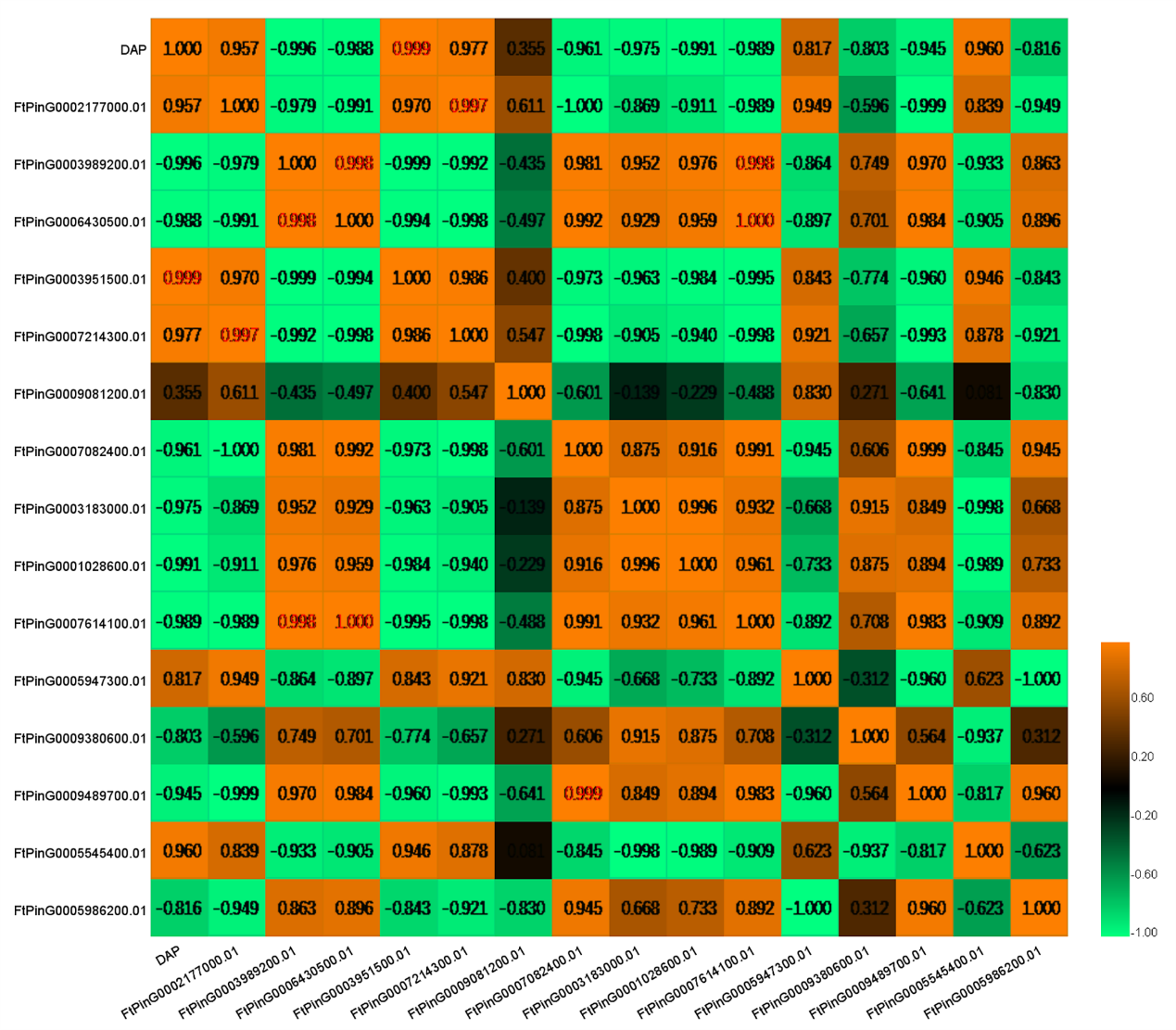
**

Supplement: Supplementary file 8 — Figure S4. The correlation between the gene expression of FtAP2s during fruit development. Positive number: positively correlated; negative number: negatively correlated. Red numbers indicate significant correlation at 0.05 levels. (DOCX 911 kb) [file 12870_2019_1681_MOESM8_ESM.docx]

**Figure S5**

**
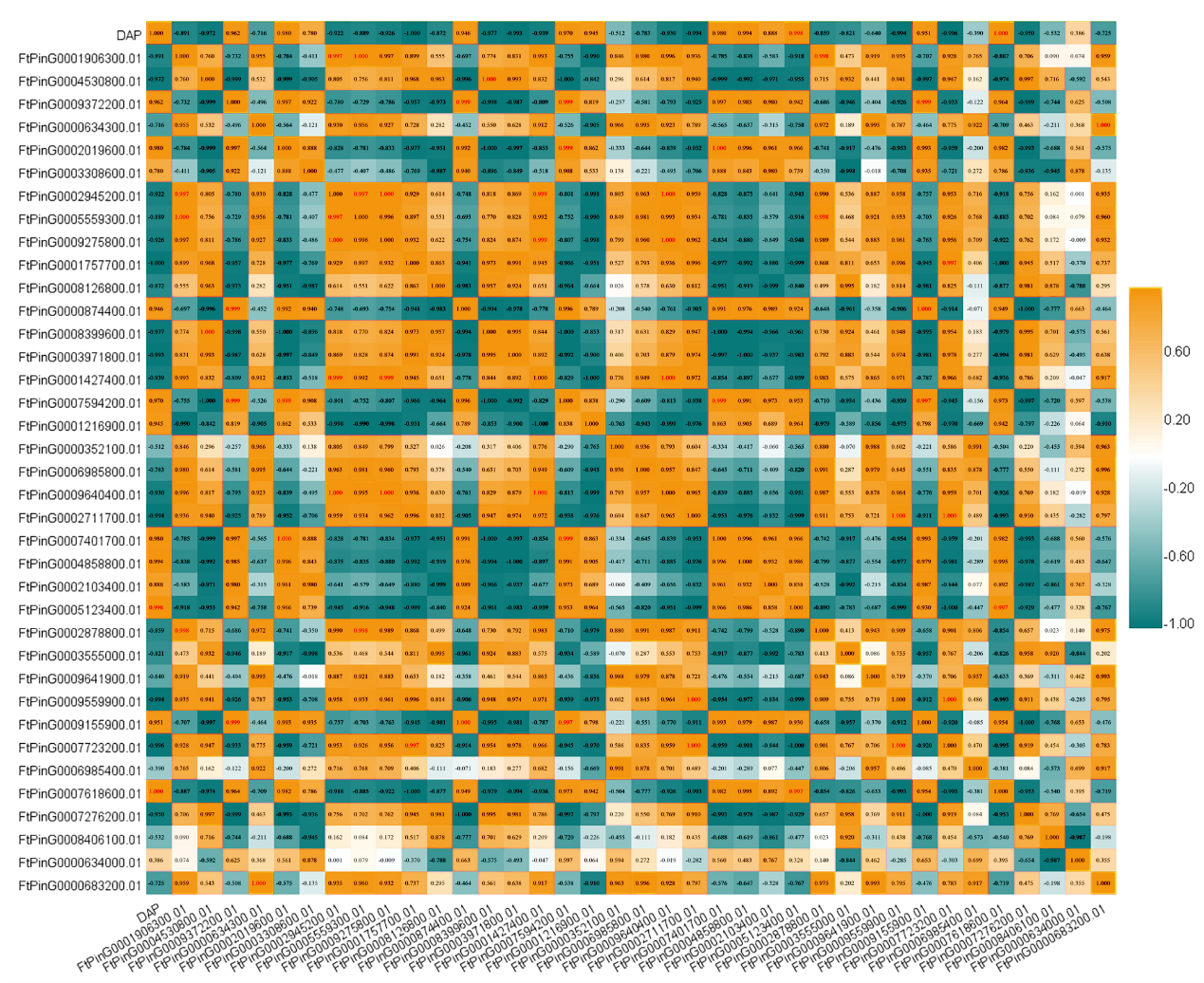
**

Supplement: Supplementary file 9 — Figure S5. The correlation between the gene expression of FtERFs during fruit development. Positive number: positively correlated; negative number: negatively correlated. Red numbers indicate significant correlation at 0.05 levels. (DOCX 1390 kb) [file 12870_2019_1681_MOESM9_ESM.docx]

**Figure S6**


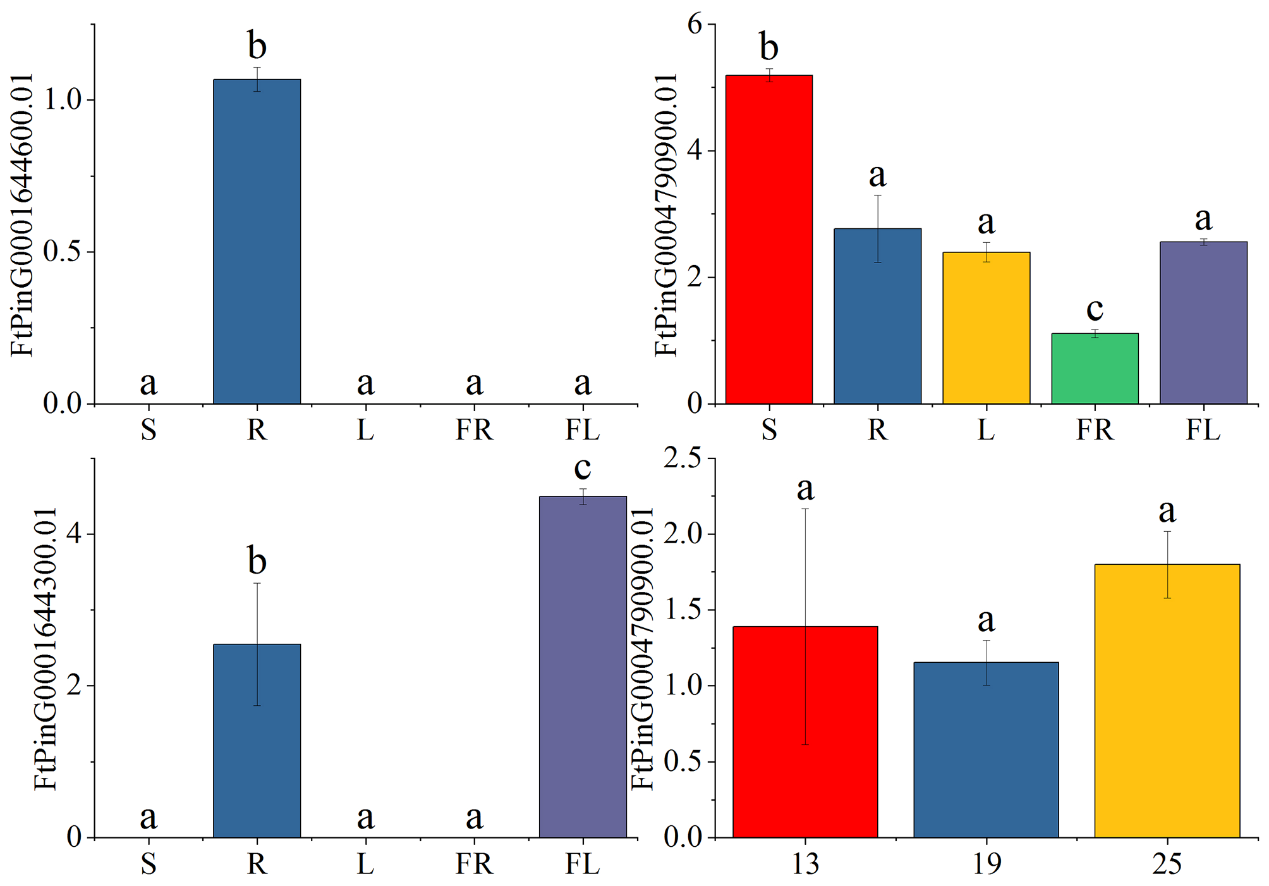

Supplement: Supplementary file 10 — Figure S6. Tissue-specific gene expression of 3 tandem duplication tartary buckwheat ERF genes. The expression patterns of 3 tandem duplication tartary buckwheat ERF genes in flower, leaf, root, stem and different stage fruit tissues were examined by qPCR. Error bars were obtained from three measurements. Small letter(s) above the bars indicate significant differences (α = 0.05, LSD) among the treatments. (DOCX 238 kb) [file 12870_2019_1681_MOESM10_ESM.docx]
